# NANS-CDG: Delineation of the Genetic, Biochemical, and Clinical Spectrum

**DOI:** 10.3389/fneur.2021.668640

**Published:** 2021-06-07

**Authors:** Bibiche den Hollander, Anne Rasing, Merel A. Post, Willemijn M. Klein, Machteld M. Oud, Marion M. Brands, Lonneke de Boer, Udo F. H. Engelke, Peter van Essen, Sabine A. Fuchs, Charlotte A. Haaxma, Brynjar O. Jensson, Leo A. J. Kluijtmans, Anna Lengyel, Klaske D. Lichtenbelt, Elsebet Østergaard, Gera Peters, Ramona Salvarinova, Marleen E. H. Simon, Kari Stefansson, Ólafur Thorarensen, Ulrike Ulmen, Karlien L. M. Coene, Michèl A. Willemsen, Dirk J. Lefeber, Clara D. M. van Karnebeek

**Affiliations:** ^1^Department of Pediatric Metabolic Diseases, Emma Children's Hospital, Amsterdam University Medical Center, Amsterdam, Netherlands; ^2^Department of Pediatric Metabolic Diseases, Amalia Children's Hospital, Radboud University Medical Center, Nijmegen, Netherlands; ^3^United for Metabolic Diseases, Amsterdam, Netherlands; ^4^Department of Neurology, Donders Institute for Brain, Cognition and Behavior, Radboud University Medical Center, Nijmegen, Netherlands; ^5^Translational Metabolic Laboratory, Department of Laboratory Medicine, Radboud University Medical Center, Nijmegen, Netherlands; ^6^Department of Radiology and Nuclear Medicine and Anatomy, Radboud University Medical Centre, Nijmegen, Netherlands; ^7^Department of Human Genetics, Donders Institute for Brain, Cognition and Behavior, Radboud University Medical Center, Nijmegen, Netherlands; ^8^Radboudumc Technology Center Clinical Studies, Radboud University Medical Center, Nijmegen, Netherlands; ^9^Department of Pediatric Metabolic Diseases, Wilhelmina Children's Hospital, University Medical Center Utrecht, Utrecht, Netherlands; ^10^Department of Pediatric Neurology, Amalia Children's Hospital, Radboud University Medical Center, Nijmegen, Netherlands; ^11^Decode Genetics/Amgen, Inc., Reykjavik, Iceland; ^12^2nd Department of Pediatrics, Semmelweis University, Budapest, Hungary; ^13^Department of Genetics, University Medical Center Utrecht, Utrecht, Netherlands; ^14^Department of Clinical Genetics, Rigshospitalet, Copenhagen University Hospital, Copenhagen, Denmark; ^15^Department of Clinical Medicine, University of Copenhagen, Copenhagen, Denmark; ^16^Department of Rehabilitation Medicine, Radboud University Medical Center, Nijmegen, Netherlands; ^17^Department of Pediatrics, University of British Columbia, Vancouver, BC, Canada; ^18^BC Children's Hospital Research Institute, The University of British Columbia, Vancouver, BC, Canada; ^19^Faculty of Medicine, School of Health Sciences, University of Iceland, Reykjavik, Iceland; ^20^Department of Pediatrics, Children's Medical Center, Landspitali–The National University Hospital of Iceland, Reykjavík, Iceland; ^21^Department of Pediatrics, Sana Klinikum Lichtenberg, Berlin, Germany

**Keywords:** congenital disorder of glycosylation, glycosylation, sialic acid biosynthesis, N-acetyl-D-neuraminic acid, skeletal dysplasia, metabolic disease, intellectual developmental disorder/IDD, thrombocytopenia

## Abstract

**Background:** NANS-CDG is a recently described congenital disorder of glycosylation caused by biallelic genetic variants in *NANS*, encoding an essential enzyme in *de novo* sialic acid synthesis. Sialic acid at the end of glycoconjugates plays a key role in biological processes such as brain and skeletal development. Here, we present an observational cohort study to delineate the genetic, biochemical, and clinical phenotype and assess possible correlations.

**Methods:** Medical and laboratory records were reviewed with retrospective extraction and analysis of genetic, biochemical, and clinical data (2016–2020).

**Results:** Nine NANS-CDG patients (nine families, six countries) referred to the Radboudumc CDG Center of Expertise were included. Phenotyping confirmed the hallmark features including intellectual developmental disorder (IDD) (*n* = 9/9; 100%), facial dysmorphisms (*n* = 9/9; 100%), neurologic impairment (*n* = 9/9; 100%), short stature (*n* = 8/9; 89%), skeletal dysplasia (*n* = 8/9; 89%), and short limbs (*n* = 8/9; 89%). Newly identified features include ophthalmological abnormalities (*n* = 6/9; 67%), an abnormal septum pellucidum (*n* = 6/9; 67%), (progressive) cerebral atrophy and ventricular dilatation (*n* = 5/9; 56%), gastrointestinal dysfunction (*n* = 5/9; 56%), thrombocytopenia (*n* = 5/9; 56%), and hypo–low-density lipoprotein cholesterol (*n* = 4/9; 44%). Biochemically, elevated urinary excretion of *N*-acetylmannosamine (ManNAc) is pathognomonic, the concentrations of which show a significant correlation with clinical severity. Genotypically, eight novel *NANS* variants were identified. Three severely affected patients harbored identical compound heterozygous pathogenic variants, one of whom was initiated on experimental prenatal and postnatal treatment with oral sialic acid. This patient showed markedly better psychomotor development than the other two genotypically identical males.

**Conclusions:** ManNAc screening should be considered in all patients with IDD, short stature with short limbs, facial dysmorphisms, neurologic impairment, and an abnormal septum pellucidum +/– congenital and neurodegenerative lesions on brain imaging, to establish a precise diagnosis and contribute to prognostication. Personalized management includes accurate genetic counseling and access to proper supports and tailored care for gastrointestinal symptoms, thrombocytopenia, and epilepsy, as well as rehabilitation services for cognitive and physical impairments. Motivated by the short-term positive effects of experimental treatment with oral sialic, we have initiated this intervention with protocolized follow-up of neurologic, systemic, and growth outcomes in four patients. Research is ongoing to unravel pathophysiology and identify novel therapeutic targets.

## Introduction

Congenital disorders of glycosylation (CDGs) comprise a large group of genetic defects affecting the glycosylation of proteins and/or lipids. CDGs are among the most rapidly expanding group of inborn errors of metabolism (1). *N*-acetyl-d-neuraminic acid synthase (NANS)–CDG (OMIM#605202) is an autosomal recessive disorder caused by genetic variants in *NANS*. *N*-acetyl-d-neuraminic acid, more commonly known as sialic acid, is essentially found at the end of glycan chains on glycoproteins or glycolipids (2). Sialic acid is abundantly present in the central nervous system and particularly on gangliosides and neural cell adhesion molecules, playing key roles in cell migration, synaptic activity, neural path finding, neurite outgrowth, and regeneration. In normal neural development, sialylated structures are highly demanded, implying that sialic acid belongs to the essential nutrients in early brain development (3). Furthermore, previous studies have identified that sialic acid plays a role in inflammatory processes, reactive oxygen species neutralization, psychiatric disorders, and neurodegeneration (2, 4).

In 2016, NANS-CDG was first described by Van Karnebeek et al. as a human inherited metabolic disease (5). The phenotypic spectrum of the reported cases included intellectual developmental disorder (IDD) (6, 7) with delay in developmental milestones, short stature with short limbs, and neurologic impairment. The case series provided the first evidence for the association between *NANS* expression and impaired brain and skeletal development. Furthermore, accumulation of *N*-acetylmannosamine (ManNAc), an upstream metabolite of sialic acid, in urine and plasma was found, providing a valuable diagnostic biomarker that can be applied for functional evaluation of pathogenicity of *NANS* variants.

Benefits of oral supplementation of sugars have been reported for several CDG subtypes, resulting in, e.g., trials with oral galactose supplementation in SLC35A2-CDG (OMIM#300896) (8). The characterization of disease-causing variants in NANS-CDG led to the identification of sialic acid as a potential therapeutic option. Research, in particular animal studies, provided evidence on the bioincorporation of dietary sialic acid in tissues and particularly into the brain (4). In parallel, knockout *nansa* zebrafish embryos were supplemented with nutrition-derived sialic acid in the early embryonic phase and showed partial rescue of the brain and skeletal phenotype (5). Clinical trials on the safety and efficacy of sialic acid reported an acceptable safety and tolerability profile in patients with GNE myopathy (OMIM#605820) (9, 10). Currently, experimental trials are running to determine whether sialic acid could serve as a valid therapeutic option in NANS deficiency.

The limited numbers of patients with NANS-CDG have hindered characterization of the phenotypic spectrum associated with *NANS* variants. More importantly, previously reported NANS-CDG patients involved only one pediatric case (5). Here, we first aimed to delineate the genetic, biochemical, and phenotypic spectrum of NANS-CDG by identifying the disease history of all NANS-CDG patients from different age groups referred to our expertise center. In order to improve counseling for this disorder, we investigated possible correlations between genotypes, phenotypes, and ManNAc excretion levels of the patients. As short limbs and skeletal anomalies are such prominent features in the first NANS-CDG report (5), we provided an overview of all CDG subtypes with any type of bone abnormality. Finally, we provide the first results of experimental sialic acid supplementation in a prenatally diagnosed NANS-CDG patient, as the first exploratory evaluation of the efficacy and safety of sialic acid in NANS-CDG.

## Methods

### Patient Selection

From 2016 onward, medical, genetic, and laboratory records were retrospectively reviewed to identify newly diagnosed NANS-CDG patients who had been referred to the Radboudumc Center of expertise on Glycosylation Disorders (RCDG) for clinical, genetic, and metabolomic evaluation. All selected patients had biallelic variants in *NANS*, which were identified by genetic testing [whole-exome sequencing (WES), targeted next-generation sequencing, whole-genome sequence]. To investigate the impact of the identified genetic variants on NANS enzyme function, we measured the upstream metabolite ManNAc in urine and plasma. In addition to the newly identified NANS-CDG patients, we included the genetic, biochemical, and clinical follow-up data of one previously reported NANS-deficient case [patient 1 in this study, patient 9 in the previous publication (5)], as this patient harbors the same *NANS* variants as two cases included in this study (patients 2 and 3). The study was approved by the Medical Ethics Board of the Radboud University Medical Center (RUMC) (CMO 2021-7373).

### Data Collection

All patients provided written informed consent through their guardians for publication of their clinical information. Consent was also obtained to publish the photos shown in **Figures 2**–**4**. Data were collected retrospectively from electronic health records from medical centers in Europe and Canada (British Columbia Children's Hospital, Vancouver, Canada; Copenhagen University Hospital, Copenhagen, Denmark; Sana Klinikum Lichtenberg, Berlin, Germany; Semmelweis University Hospital, Budapest, Hungary; National University Hospital of Iceland, Reykjavík, Iceland; RUMC, Nijmegen, the Netherlands) via the referring physicians, including available data until November 2020 or until start of sialic acid treatment. The neuroradiological and skeletal images were reassessed to ensure uniform analysis of brain magnetic resonance imaging (MRI) scans and X-rays of the skeleton. Additional data were obtained via the Nijmegen Pediatric CDG Rating Scale (NPCRS) questionnaire, evaluating disease severity and longitudinal natural history of CDG patients (11).

### Severity Score

To evaluate the severity of disease, we used two scoring systems to determine the overall severity score per case. First, we calculated an individual severity score based on the NPCRS, by adding scores together for each patient (11). Zero (lowest score) indicates a mild phenotype; 110 (highest score) indicates a severe phenotype. The NPCRS is subdivided into three age ranges, according to the developmental phases, including infancy and early childhood (0–24 months), middle childhood (2–11 years), and adolescence (12–18 years). The NPCRS was designed to follow CDG patients longitudinally and to capture the most common symptoms (rare symptoms were excluded from the scale). Congenital brain abnormalities and skeletal dysplasia appear to be important factors in determining the severity of clinical symptoms. However, neither is included in the NPCRS, and consequently, a different approach and scoring system were needed: the clinical severity classification. We adopted brain and skeletal abnormalities in the clinical severity classification. Assessment of the brain MRI scans and X-rays of the bones was performed by experts in neuroradiology and pediatric radiology, and the abnormalities were classified into mild, moderate, or severe. The clinical severity classification was determined per affected case, rated by two independent specialists, based on the NPCRS score completed with the presence and severity of the brain abnormalities and skeletal dysplasia.

### Biochemical Analysis

Quantitative proton nuclear magnetic resonance (^1^H NMR) spectroscopy was performed to quantify excretion of ManNAc in patients' urinary samples. Next-generation metabolomic screening (NGMS), which encompasses untargeted metabolomics analysis using quadrupole time-of-flight mass spectrometry, was performed in patients' plasma samples to semiquantify the increase in ManNAc, based on a fold change in intensity in patients vs. controls. The NGMS method was introduced and clinically validated at the translational metabolic laboratory at the RUMC in Nijmegen. For a detailed description, see the publication of Coene et al. (12).

### Statistical Analysis

Correlations between biochemical and clinical phenotype were assessed using the Pearson linear correlation coefficient *r*, double-sided. *p* < 0.05 was considered statistically significant.

### Experimental Treatment With Sialic Acid

The study included a newborn male (patient 2) who had been prenatally diagnosed with NANS deficiency via WES using extracted DNA from uncultured amniotic fluid cells at 31 weeks of gestation. With approval of the Board of Directors of the Radboudumc and informed consent of both parents, maternal experimental treatment with oral sialic acid (manufactured by Jennewein Biotechnologie GmbH), at a dosage of 1.500 mg four times a day, was started at 34 weeks of gestation. This dose selection was based on previous clinical trials with GNE-myopathy (OMIM#605820) patients (9, 10). The treatment was started and monitored according to the experimental treatment protocol. During the prenatal treatment period, advanced ultrasound imaging was performed to monitor prenatal growth every 2 weeks. Sialic acid passes into breast milk (2). After birth, the patients' mother continued with sialic acid to treat the patient via breastfeeding. At the age of 12 days, maternal treatment was stopped, and the infant was given sialic acid in a dose of 4,000 mg/m^2^ per day in four doses. First weekly, then monthly, the dosage was corrected for body surface area. The following outcomes were evaluated: somatic growth, development, neurologic features and movement scale, low-density lipoprotein (LDL) cholesterol level, and thrombocyte count.

## Results

### Patient Screening and Identification

In total, nine patients were referred to the RCDG with suspicion of NANS-CDG based on *NANS* variants identified by genetic testing. Of these, eight patients were newly referred, and one patient (patient 1) was previously reported (5). We here report the follow-up of the nine patients (five male and four female patients) from nine families and six countries (Canada, Denmark, Germany, Hungary, Iceland, and the Netherlands). The median age of the patients was 7 years (range = 3 months to 28 years). All patients were born to non-consanguineous parents. Family history included oculocutaneous albinism, vanishing twin and two spontaneous miscarriages, hyperhomocysteinemia and Noonan syndrome.

### Genotypic Spectrum

**Figure 1:**
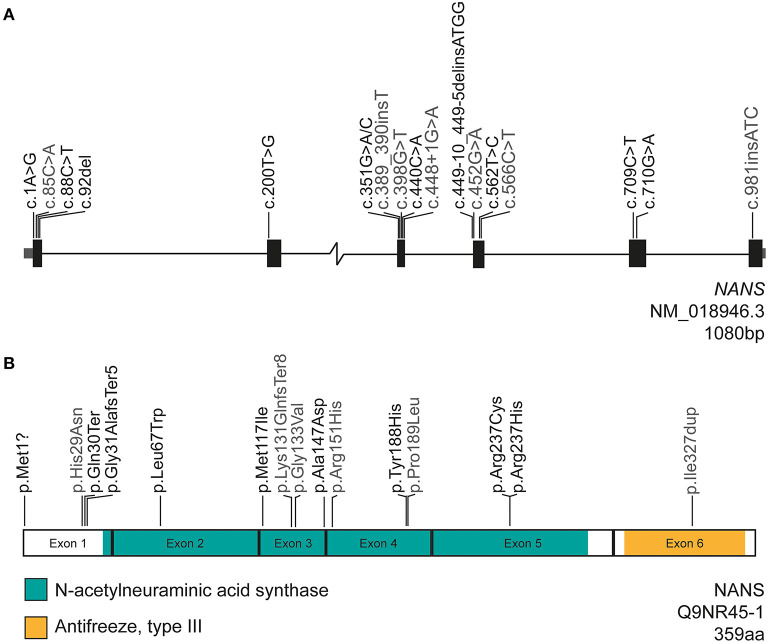
Gene and protein structure of *NANS* encoding NANS. The nature and position of the variants reported in this study (black) and the previous case study (gray) are indicated (5). **(A)** *NANS* is 1,080 bp in length and consists of six exons. In total, 17 variants associated with NANS-CDG are reported in both studies combined, of which 10 are newly described in this case study. **(B)** The NANS protein is 359 amino acids in length and contains two domains: the N-term, the *N*-acetylneuraminic acid synthase domain (green) and the C-term antifreeze, type III domain (orange). The majority of variants are located in a domain from which 10 are located in the *N*-acetylneuraminic acid synthase domain.

The biallelic variants in *NANS* associated with NANS-CDG are listed in Table 1 and mapped in Figure 1. In nine patients, we detected eight compound heterozygous variants and one homozygous variant. From the total of 11 monoallelic variants, eight variants were novel: c.1A>G p.(Met1?), c.88C>T p.(Gln30^*^), c.92del p.(Gly31Alafs^*^5), c.200T>G p.(Leu67Trp), c.351G>A p.(Met117Ile), c.922_925dup p.(Met309Asnfs^*^11), c.440C>A p.(Ala147Asp), and c.710G>A p.(Arg237His). The combined annotation-dependent depletion (CADD) score was >18 for all variants and >24 for all coding variants, which suggests a pathogenic effect of the variants on protein function. The genomic position of each variant and the results from different prediction tools are summarized in Table 1. The deletion insertion detected in patient 5, c.449–10_449–5delGATTACinsATGG, was previously reported and shown to lead to aberrant splicing of exons 3 and 4 (5). Two patients (patients 2 and 3) harbored the same compound heterozygous variants as patient 9 from the first case series from 2016 (5): c.709C>T p.(Arg237Cys); c.562T>C p.(Tyr188His). These three patients were all of Dutch ancestry suggesting common founders.

**Table 1:** Overview of demographic, genomic, biochemical, and clinical data of nine patients with NANS-CDG.

**Demographics**	**Patient 1**	**Patient 2**	**Patient 3**	**Patient 4**	
Ancestry	Canadian/European (the Netherlands)	European (the Netherlands)	European (the Netherlands)	European (Hungary)	
Gender	Male	Male	Male	Female	
Age at evaluation	8 years	1–3 months	2 years	10 months	
Age molecular diagnosis	3 years	Prenatal (31 weeks 0 days)	6 months	10 months	
**Genotypic spectrum**					
NANS DNA variants	c.709C>T; c.562T>C	c.709C>T; c.562T>C	c.709C>T; c.562T>C	c.709C>T; c.440C>A	
Genomic DNA position^a^	g.100843203; g.100840588	g.100843203; g.100840588	g.100843203; g.100840588	g.100843203; g.100839291	
Variant type (protein effect)	Missense; missense	Missense; missense	Missense; missense	Missense; missense	
Amino acid change	p.(Arg237Cys); p.(Tyr188His)	p.(Arg237Cys); p.(Tyr188His)	p.(Arg237Cys); p.(Tyr188His)	p.(Arg237Cys); p.(Ala147Asp)	
gnomAD frequency^b^	0.00001314; 0.00003291	0.00001314; 0.00003291	0.00001314; 0.00003291	0.00001314; 0.00001973	
PolyPhen score^b^	Probably damaging; probably damaging	Probably damaging; probably damaging	Probably damaging; probably damaging	Probably damaging; probably damaging	
SIFT score^b^	Deleterious; deleterious	Deleterious; deleterious	Deleterious; deleterious	Deleterious; deleterious	
ClinVar^b^	Pathogenic; pathogenic	Pathogenic; pathogenic	Pathogenic; pathogenic	Pathogenic; unknown	
CADD score^c^	32; 27.2	32; 27.2	32; 27.2	32; 29.9	
**Biochemical spectrum**					
ManNAc excretion in urine (μmol/mmol creatinine, ref 0 μmol/mmol creatinine)	295^e^	330; 297^f^	405; 520^g^	530^h^	
ManNAc increase in plasma (fold change in NGMS vs. controls)	11×	25×	10×	Plasma not available	
**Phenotypic spectrum**					
Birth parameters					
Gestational age (weeks + days)	41+3	40+5	39+3	37+0	
Birth weight (g)	2,865 (P4)	3,000 (P7)	3,228 (P23)	2,190 (P < 2.5)	
Birth height (cm)	47 (P5)	NR	46 (P2)	40 (<P2)	
Head circumference (cm)	NR	32 (<P2.5)	35 (P82)	29 (<P2.5)	
Apgar scores (after 1, 5, 10 min)	NR	6, 8, 9	3, 8, 10	9, 10, 10	
Neonatal jaundice	Y (phototherapy)	Y	Y (phototherapy)	Y	
Respiratory distress	N	N	Y	N	
Complications at birth	Skeletal dysplasia	Hypotonia; skeletal dysplasia; petechiae; polycythemia; metabolic compensated respiratory acidosis; thrombocytopenia	Axial hypotonia; skeletal dysplasia; hyperlaxity; dysfunctional sucking	Skeletal dysplasia; hydrocephalus	
Interventions at birth	N	Tube feeding	Vit. K supp.; short-term oxygen support; tube feeding	N	
**Psychomotor development and cognition**					
Intellectual disability	+++(home schooled, nurse participates in care)	NA	NA	NA	
IQ range	NR	NA	NA	NA	
Adaptive functioning	Delayed	NA	Delayed	Delayed	
Global developmental delay	++++	+	+++	++	
Gross motor skills	Delayed, no ambulation	NA	Delayed (according to Bayley scale), no ambulation	Delayed	
Fine motor skills	Delayed (unspecified)	NA	Delayed (according to Bayley Scale)	NR	
Language development	No speech	NA	Delayed	NA	
**Neurologic symptoms**					
Seizure history	Y	N	N	Y	
Abnormalities of muscle tone	Generalized hypotonia	Axial hypotonia	Axial hypotonia; limb hypertonia	Axial, limb hypotonia	
Muscle strength	Inadequate	Normal	Inadequate around hips	Inadequate	
Reflexes	NR	NR	Absence of menace reflex	NR	
Ataxia	NR	NR	N	N	
Cranial nerves	Intact	NR	NR	NR	
Other neurological abnormalities	Hydrocephalus	N	Dystonia; spasticity; tremor	West syndrome	
Neuroimaging (MRI)	**At age 4 months−3.5 years:** Ventriculomegaly; Abnormality in periventricular white matter; Cerebral atrophy; Persistent vacuum vergae; Abnormal basal nuclei; Hypoplasia of the corpus callosum and splenium, aplasia rostrum; Asymmetry of cerebellum	**Prenatal:** Hypoplastic cerebellum; Periventricular pseudocysts; Polymicrogyria **Postnatal (2 days):** Ventriculomegly; Cerebral atrophy; Widened cisternae and/or sulci; Hypoplastic brainstem; Hypoplastic cerebellum; Periventricular pseudocysts; Cavum septum pellucidum; Cerebellar hemorrhage; Abnormal corpus callosum with hypoplastic splenium	**Prenatal:** Suspected hypoplastic cerebellum; Small cavum septum pellucidum; Mild ventriculomegaly; Suspected syntelencephaly **Postnatal (4 days):** Small but anatomically normal cerebellum; Moderate ventriculomegly; Limited volume of corpus callosum and periventricular white matter; Small optic chiasm; Absence of septum pellucidum; Suggestion of cortical malformation of the left temporoparietal region **At 2 years:** Progressive white > gray matter atrophy; Severe ventriculomegaly; Aqueduct stenosis	**At 8 months:** Enlarged lateral ventricles and third ventricle; Cerebral atrophy; Absent splenium of the corpus callosum; Aqueduct stenosis	
**Somatic growth**					
	*Age 8.5 years*	*3 months*	*Age 26 months*	*Age 10 months*	
Length (cm)	91 (<P0.01)	56 (P0.4)	76.2 (P0.01)	57 (<P0.01)	
Weight (kg)	19.8 (P0.5)	5.435 (P8)	9.426 (P0.01)	5.3 (<P0.01)	
Head circumference (cm)	NR	41 (P85)	48.3 (P31)	39 (<P0.01)	
Short stature	Y	Y	Y	Y	
**Bones**					
Skull^d^	Frontal bossing	NR	Frontal bossing	NR	
Spine^d^	Flat vertebra; Irregular calcification of the endplates; Scoliosis and lumbar kyphosis	NR	L2 ventroapical deformity, resulting in kyphosis and scoliosis; Abnormal ossification centers (CT)	NR	
Pelvis^d^	Sclerosis iliac crest; Flat acetabular roofs; Shallow acetabula; Short femoral neck	NR	Small iliac wings; Shallow flat acetabula; Small femoral head epiphysis; Short femoral neck; Luxated left hip	NR	
Limbs^d^	*Upper extremities*: Metaphyseal widening distal ulna and radius *Lower extremities*: NR	*Upper extremities*: Metaphyseal widening; Sclerosis; Irregularity *Lower extremities:* Metaphyseal widening; Sclerosis; Irregularity	*Upper extremities*: Metaphyseal widening; Sclerosis; Irregularity *Lower extremities:* Metaphyseal widening; Sclerosis; Irregularity	*Upper extremities*: Metaphyseal widening; Sclerosis; Irregularity; Small flat epiphysis distal radius *Lower extremities*: Metaphyseal widening; Sclerosis; Irregularity; Genu vara	
Joint hypermobility	NR	NR	Y	N	
**External features**					
Facial dysmorphisms	Y	Y	Y	Y	
Skin anomalies	N	N	N	N	
**Eye**					
	Strabismus; amblyopia; myopia; cone-red dystrophy; visual impairment	Strabismus	Delayed visual development	Strabismus	
**Heart**					
	Dilated aortic root and abdominal aorta	Normal	Normal	Normal	
**Auditory**					
	Conductive hearing loss	Normal	Normal	Abnormal BERA test 1 side	
**Gastrointestinal and nutrition**					
Constipation	+++	N	+++	Y	
Abdominal distention	++	+	+++	Y	
Other pathology	GastroesophageaI reflux; G-J tube *in situ*; partial bowel obstruction	Anus perforatus	N	N	
**Urinary tract**					
Urinary tract	Neurogenic bladder	NR	Normal	Urethral stenosis; hydronephrosis; hydroureter	
External genitalia development	NR	Normal	Normal	Normal	
Onset of puberty	NA	NA	NA	NA	
Immunologic (recurrent infections)					
	Y	N	Y	Y	
**Other striking features**					
	Obstructive sleep apnea	Positional preference right; Flattened aspect hands	Positional preference right; Excessive mucus production	Excessive mucus production	
**Clinical severity**					
Clinical severity classification	Profound	Mild-moderate	Profound	Profound	
NPCRS	48	13	40	38	
**Laboratory findings**					
**Hematology**					
Anemia	N	N	N	Y	
Thrombocytopenia	Y	Y	Y	Y	
**Biochemistry**					
Lactate	NR	Normal	↑(Recovered)	NR	
Electrolytes	Normal	Normal	Normal	Normal	
Kidney function	Normal	Normal	Normal	Normal	
Liver/biliary function	↓Alkaline phosphatase	↑Alkaline phosphatase	↑Alkaline phosphatase	↑Alkaline phosphatase	
Lipids	NR	↓LDL	↓LDL	Normal	
Endocrine	Normal	NR	Normal	Normal	
**Demographics**	**Patient 5**	**Patient 6**	**Patient 7**	**Patient 8**	**Patient 9**
Ancestry	European (Germany/Russia)	European (Iceland)	European (Dutch)	European (Denmark)	European (Dutch)
Gender	Female	Female	Male	Male	Female
Age at evaluation	7 years	13 years	16 years	17 years	28 years
Age molecular diagnosis	6 years	10 years	11 years	15 years	25 years
**Genotypic spectrum**					
NANS DNA variants	c.1A>G; c.449–10_449–5delGATTACinsATGG	c.351G>A; c.922_925dup	c.710G>A; c.92del	c.88C>T; c.200T>G	c.709C>T homozygous
Genomic DNA position^a^	g.100819091; g.100840465	g.100839202; g.100845179_100845182dup	g.100843204; g.100819182	g.100819178; g.100823131	g.100843203
Variant type (protein effect)	Start_lost; splice-region_variant; intron_variant	Missense; frameshift	Missense; frameshift	Stop_gain; missense	Missense
Amino acid change	p.(Met1?); unknown	p.(Met.117IIe); p.(Met309Asnfs*11)	p.(Arg237His); p.(Gly31Alafs*5)	p.(Gln30*); p.(Leu67Trp)	p.(Arg237Cys)
gnomAD frequency^b^	0.000006595; unknown	0.001302; 0.000007954^i^	0.000006570; 0.00001314	Unknown; 0.00001314	0.00001314
PolyPhen score^b^	Benign; unknown	Benign; unknown	Possibly damaging; unknown	Unknown; benign	Probably damaging
SIFT score^b^	Deleterious_low_confidence; unknown	Tolerated; unknown	Deleterious; unknown	Unknown; deleterious	Deleterious
ClinVar^b^	Unknown; pathogenic	Benign; unknown	Unknown; unknown	Unknown; unknown	Pathogenic
CADD score	24; 18.01	24.3; 23.2	29.5; 32	41; 25	32
**Biochemical spectrum**					
ManNAc excretion in urine (μmol/mmol creatinine, ref 0 μmol/mmol creatinine)	Urine not available	46^j^	45^k^	10^l^	Urine not available
ManNAc increase in plasma (fold change in NGMS vs. controls)	Plasma not available	Plasma not available	2×	3×	6×
**Phenotypic spectrum**					
**Birth parameters**					
Gestational age (weeks + days)	35+0	Full term	40+5	40+0	41+3
Birth weight (g)	1,550 g (<P2.5)	3,000 (P8)	3,620 (P48)	4,560 (P97)	3,920 (P82)
Birth height (cm)	NR	48 (P25)	50 (P50)	NR	48 (P25)
Head circumference (cm)	NR	36 (P95)	NR	NR	37 (P98)
Apgar scores (after 1, 5, 10 min)	NR	NR	Unremarkable	4, 5, 6	10, 10, 10
Neonatal jaundice	NR	N	NR	N	Y
Respiratory distress	N	N	Y	N	N
Complications at birth	N	Skeletal dysplasia	NR	Low blood pressure; hypotonia	Skeletal dysplasia; recurrent choking
Interventions at birth	Tube feeding; care at the NICU (prematurity)	N	Short-term oxygen support	Intravenous saline	NR
**Psychomotor development and cognition**					
Intellectual disability	+++(attending special school)	+++(attending special school, autism)	+(attending special school)	+(attending special school)	+++(lives in nurse home)
IQ range	NR	20–40 (age and test unspecified)	60–75 (according to WISC-III-NL)	56 (age 6, test unspecified)	NR
Adaptive functioning	Delayed	Delayed	Delayed	Delayed	Delayed
Global developmental delay	+++	+++	+	+	++++
Gross motor skills	Unaided ambulation	Unaided ambulation, mild mobility problems	Unaided ambulation	Unaided ambulation	Delayed, no ambulation
Fine motor skills	NR	NR	Delayed (unspecified)	Delayed (unspecified)	Delayed (unspecified)
Language development	Delayed	No speech	Delayed	Delayed, dyspraxia	No speech
**Neurologic**					
Seizure history	N	N	N	N	Y
Abnormalities of muscle tone	Hypotonia	Hypotonia	Generalized hypotonia	Hypotonia	Generalized hypertonia
Muscle strength	Mild weakness	Mild weakness	Normal	NR	Inadequate
Reflexes	NR	NR	Hyperreflexia	NR	Low-normal
Ataxia	Y (mild)	Y (mild)	N	N	Y
Cranial nerves	NR	NR	NR	NR	NR
Other	N	N	NR	N	N
Neuroimaging (MRI)	**At 2 years:** Persistent cavum septum pellucidum	**At 11 years:** CT scan: normal	**At 12 years:** Cavum septum pellucidum and vergae	NR	**At 9 months:** Ventriculomegaly **At 3 years:** Cerebral atrophy; widened cisternae and/or sulci; atrophy of the caudate nucleus; **At 15 years:** Ventriculomegaly; cavum septum pellucidum
**Somatic growth**					
	*Age 7 years*	*Age 8.5 years*	*Age 16 years*	*Age 13.5 years*	*Age 26 years*
Length (cm)	105.6 (<P0.01)	115 cm (<P0.01)	151.3 cm (P0.3)	160.6 (P44)	126 (<P0.01)
Weight (kg)	17.2 (P0.5)	20 kg (P0.3)	47.3 kg (P5)	70 (P95)	30 (<P0.01)
Head circumference (cm)	50 (P10)	NR	55 cm (P80)	NR	58 (>P95)
Short stature	Y	Y	Y	N	Y
**Bones**					
Skull^d^	NR	NR	NR	NR	NR
Spine^d^	Vertebral plates sclerosis from dorsal to ventral over time; Increased lumbar lordosis	Vertebral plates abnormal sclerosis from dorsal to ventral over time, resulting in wavy double contour plates	Double contour of the vertebral plates with less calcification anterior	NR	Scoliosis
Pelvis^d^	NR	Small iliac wings; Small femoral heads and neck	Small iliac wings, dysplastic acetabula; Small femoral heads and neck	NR	Small femoral head and neck
Limbs^d^	*Upper extremities*: Metaphyseal widening; Sclerosis; Irregularity distal ulna and radius *Lower extremities*: NR	*Upper extremities*: Metaphyseal widening; Sclerosis; Irregularity distal ulna and radius *Lower extremities*: Metaphyseal irregular striated sclerosis; Dysplastic knee joints; Fibular overgrowth cf. tibia	*Upper extremities*: Subtle metaphyseal widening and sclerosing *Lower extremities*: Subtle metaphyseal widening and sclerosing; Striated sclerosing at knee metaphysis with small irregular epiphysis	NR	*Upper extremities*: Small epiphyses *Lower extremities*: Small epiphyses
Joint hypermobility	N	N	Y	NR	Y
**External features**					
Facial dysmorphisms	Y	Y	Y	Y	Y
Skin anomalies	NR	NR	Eczema	NR	N
**Eye**					
	Mild strabismus; hyperopia	Strabismus; nystagmus	Normal	Normal	Normal
**Heart**					
	Normal	Normal	Normal	NR	Normal
Auditory					
	Normal	Normal	Normal	Normal	Normal
**Gastrointestinal and nutrition**					
Constipation	N	N	N	N	+++
Abdominal distention	N	N	NR	N	++
Other pathology	N	N	Swallowing difficulty during infancy	N	NR
**Urinary tract**					
Urinary tract	Normal	Normal	NR	NR	Bladder emptying disorder
External genitalia development	NR	NR	NR	NR	Normal
Onset of puberty	NA	No	Age-adequate	Age-adequate	NR
Immunologic (recurrent infections)					
	N	NR	Y	N	Y
**Other features**					
	N	NR	N	Overweight	Excessive mucus production
**Clinical severity**					
Clinical severity classification	Severe	Mild	Mild	Mild	Severe-profound
NPCRS	21	19	13	8	36
**Laboratory findings**					
**Hematology**					
Anemia	N	N	N	NR	Y^m^
Thrombocytopenia	N	N	N	NR	Y^n^
**Biochemistry**					
Lactate	Normal	NR	Normal	NR	↑(Recovered)
Electrolytes	Normal	Normal	Normal	NR	Normal
Kidney function	Normal	Normal	Normal	NR	Normal
Liver/biliary function	Normal	Normal	Normal	NR	Normal
Lipids	Normal	Normal	↓LDL; ↓Triglycerides	NR	↓LDL
Endocrine	Normal	Normal	Normal	NR	Normal

*+, mild; ++, moderate; +++, severe; ++++, profound; ↑, elevated; ↓, decreased; Y, yes; N, no; NR, not reported; NA, not applicable; NGMS, next-generation metabolic screening; NPCRS, Nijmegen Pediatric CDG Rating Scale*.

a *GRCh37*.

b *GnomAD v3.1, accessed December 2020, scores determined December 2020*.

c *CADD score calculated with GRCh37*.

d *Reported on X-ray*.

e *Urine sample at age 3 years*.

f *Urine samples at age 2 months and 5 months*.

g *Urine sample at age 6 months and 24 months*.

h *Urine sample at age 10 months*.

i *GnomAD v2.1.1*.

j *Urine sample at age 10 years*.

k *Urine sample at age 12 years*.

l *Urine sample at age 17 years*.

m *Due to special diet*.

n *Measured at age of 23 months, normal platelet count at age 26 and 28 years*.

### Biochemical Spectrum

For all patients in whom ManNAC excretion was measured, NANS-CDG could be confirmed on a biochemical level based on elevated ManNAc excretion in urine, as measured by ^1^H NMR spectroscopy. Considerable variation in levels of ManNAc excretion was found, ranging from 10 to 530 μmol/mmol creatinine (detection limit of ManNAc in the ^1^H NMR assay was 10 μmol/mmol creatinine; in urine of healthy controls, ManNAc cannot be detected). In all patients in whom ManNAC in plasma was measured, also increased ManNAc (vs. controls) was measured by NGMS. For two patients, ManNAc excretion in urine could not be determined at time of inclusion because of unavailability of urine samples. However, NANS-CDG diagnosis was confirmed at the genetic level.

### Phenotypic Spectrum

Detailed clinical reports of the individual cases are presented in the Supplementary File 1. An overview of the disease features is recorded in Table 1. The hallmark clinical features previously reported (5) were confirmed in the current patients: IDD, neurologic disability, and recognizable facial features in all patients, short stature, skeletal dysplasia, and short limbs in eight of nine patients (89%). We now describe the neonatal presentations of all cases, followed by previously reported hallmark clinical features and last newly observed clinical features.

#### Neonatal Presentation

**Figure 2:**
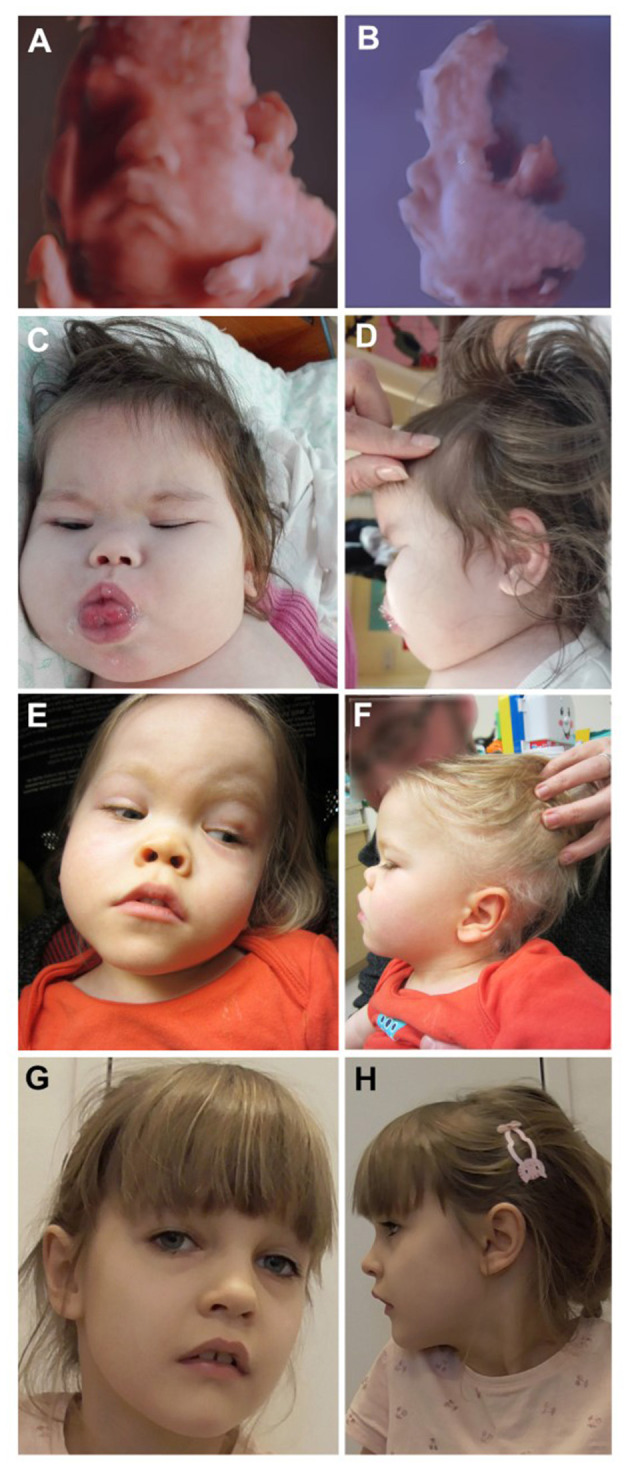
**(A)** Prenatal 3D ultrasound of patient 2 at 34 weeks 2 days' gestational age, frontal view showing carpe-shaped mouth with tenting of both upper and lower lip. **(B)** Prenatal 3D ultrasound of patient 2 at 34 weeks 2 days' gestational age, lateral view showing craniofacial features with depressed nasal bridge, upturned nasal tip and prominent upper lip. **(C)** Facial features of patient 4 at age 10 months, frontal view showing depressed midface with full cheeks and prominent philtral ridges. **(D)** Facial features of patient 4 at age 10 months, lateral view showing deep-set eyes and low-set ears. **(E)** Facial features of patient 1 at age 3 years, frontal view showing high forehead, depressed midface, full cheeks, and tented mouth. **(F)** Facial features of patient 1 at age 3 years, lateral view showing deep-set eyes, upturned nasal tip, and low-set and posteriorly rotated ears. **(G)** Facial features of patient 5 at age 7 years, frontal view showing minimal dysmorphic features with tented upper lip and widely spaced teeth. **(H)** Facial features of patient 5 at age 7 years, lateral view showing mild posterior rotated and low-set ear and prominent, short, philtrum.

Patient 2 was diagnosed prenatally; WES was performed because an ultrasound showed recognizable facial dysmorphisms and abnormal growth velocity of both brain and skeletal tissue. The prenatal MRI scan at 34 weeks 2 days of gestation showed a remarkably similar face to patient 9 of the 2016 report (5) who harbors the same *NANS* variants (Figure 2). At birth, skeletal dysplasia, more specifically a phenotype of short limbs (measured on clinical examination), was observed in five of nine patients (56%). Three additional patients were diagnosed with short limbs (based on clinical evaluation) later in life. Neonatal jaundice was observed in five of nine patients (56%), for which two patients needed phototherapy treatment. After birth, tube feeding due to prematurity and inefficient sucking and drinking skills was needed in three of nine patients (33%). In one patient (11%), a G-tube was required after 3 weeks because of failure to thrive. Three of nine patients (33%) were small for gestational age, and one patient (11%) was born prematurely. One patient (11%) experienced recurrent choking incidents since birth, without the need for tube feeding. Other reported neonatal problems were hypotonia in three of nine patients (33%), respiratory distress requiring short-term respiratory support in two of nine patients (22%), mild metabolic compensated respiratory acidosis (11%), polycythemia (11%), thrombocytopenia (11%), petechiae (11%), hyperlaxity (11%), hydrocephalus (11%), and low blood pressure (requiring intravenous saline) (11%) in one patient.

#### Known NANS-CDG Clinical Features

##### Psychomotor Development and Cognition

Early-onset global developmental delay was present in seven of nine patients (78%). The delay in reaching developmental milestones was highly variable; three of seven patients (43%) ≥2 years never achieved unaided ambulation, three of seven patients (43%) achieved unaided walking before the age of 20 months, and one patient achieved unaided walking at the age of 30 months. In two of nine patients (22%), gross motor skills such as sitting (at 8 months) and walking unaided (18–19 months) were not significantly delayed. Speech and language delay were present in all patients, with none of the patients acquiring normal speech (varying form a few sound to speech with verbal apraxia). Similarly, all patients were cognitively impaired, with all patients 5 years or older suffering from mild to severe intellectual disability. None of the patients was living independently or was completely independent for all activities of daily living.

##### Neurologic Symptoms

Muscle tone was abnormal in all patients, with hypotonia in eight of nine patients (89%) and hypertonia in two of nine patients (22%) (in one patient mixed hypotonia and hypertonia). Muscle weakness affected six of nine patients (67%). Epilepsy and ataxia were present in three of nine patients (33%); seizures were successfully controlled with antiepileptic drugs in one patient, whereas two patients suffered intractable epilepsy (for which one patient used cannabis oil).

##### Neuroimaging

**Figure 3:**
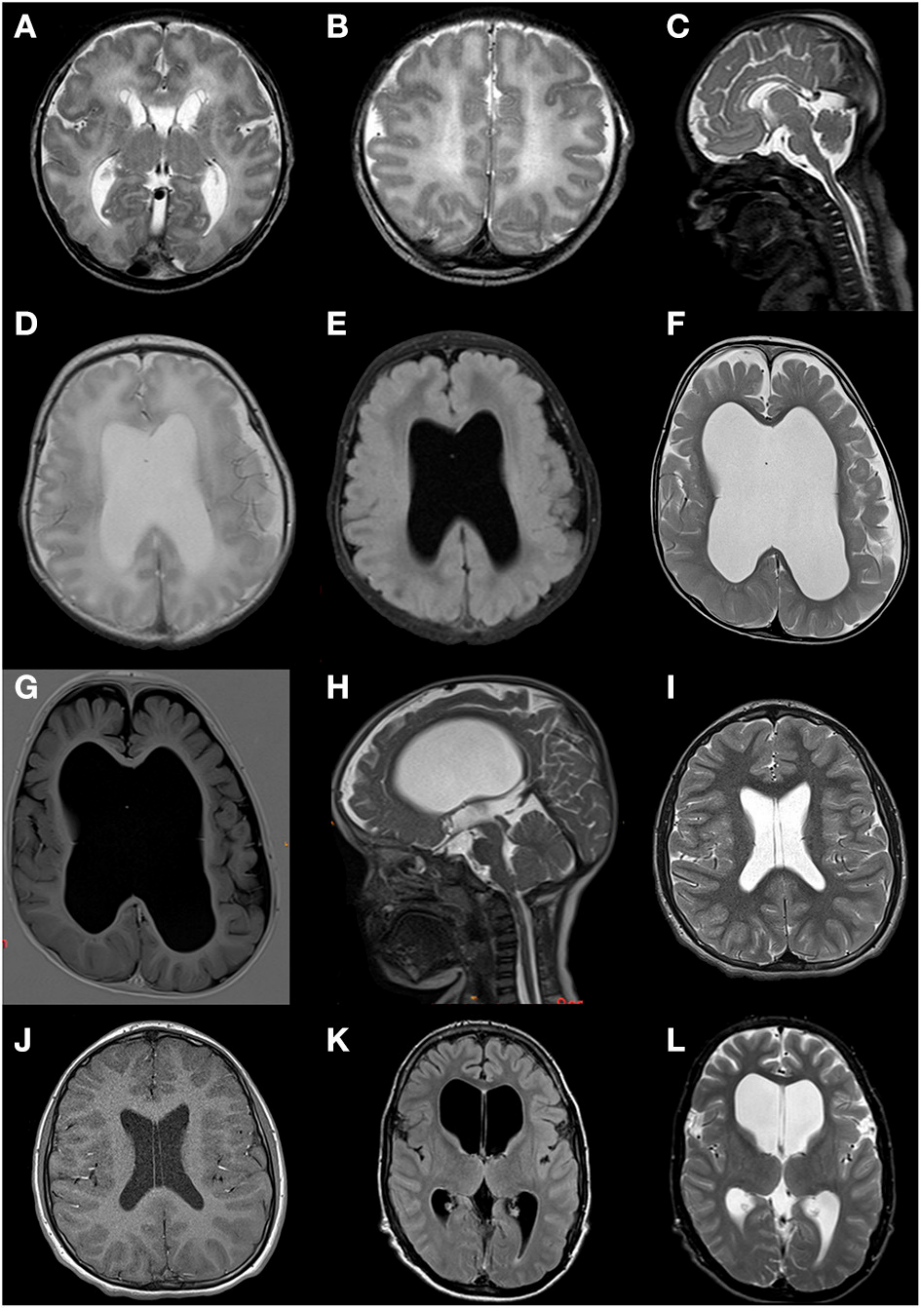
Brain MRI scans of patients 2, 3, 7, and 9. **(A–C)** Patient 2 at age 2 days; fetal gyral pattern with simplified sulcation, thin corpus callosum with hypoplastic splenium, widened ventricles and cisternae, subependymal pseudocysts, and a cavum septum pellucidum. **(D,E)** Patient 3 at age 4 days; moderate ventriculomegaly, absence of septum pellucidum, limited volume of the corpus callosum and periventricular white matter, suggestion of cortical malformation of the left temporoparietal region. **(F,G)** Patient 3 at age 25 months; ventriculomegaly (lateral and third ventricle) and enlarged subarachnoid space due to progressive loss of supratentorial white and gray matter volume; absence of septum pellucidum. **(H)** Patient 3 at age 28 months; severely enlarged lateral and third ventricle, narrow aqueduct and fourth ventricle, suggesting not only *ex vacuo* dilatation but dysfunction of cerebrospinal fluid. **(I,J)** Patient 7 at age 12 years; normal MRI but with persistent cavum septum pellucidum and vacuum vergae. **(K,L)** Patient 9 at age 15 years; marked ventriculomegaly (especially lateral ventricles).

Neuroimaging was available for eight of nine patients (CT in one patient, MRI in the other patients), 75% of whom showed abnormalities as illustrated in Figure 3. Striking features present in six of eight patients (75%) included an abnormal septum pellucidum, in five of eight patients (63%) ventriculomegaly, and in five of eight patients (63%) cerebral atrophy. Further reported findings were hypoplasia of the corpus callosum and/or splenium (*n* = 4/8; 50%), hypoplasia/asymmetry of the cerebellum (*n* = 2/8; 38%), abnormal cisternae and/or sulci (*n* = 2/8; 25%), aqueduct stenosis (*n* = 2/8; 38%), abnormal basal ganglia (*n* = 2/8; 25%), hypoplastic brainstem (*n* = 1/8; 25%), abnormal periventricular white matter (*n* = 1/8; 13%), periventricular pseudocysts (*n* = 1/8; 13%), polymicrogyria (*n* = 1/8; 13%), cerebellar hemorrhage (*n* = 1/8; 13%), and a small optic chiasm (*n* = 1/8; 13%) (Figure 3). In patient 3, the only individual in whom sequential imaging was done, progressive enlargement of ventricles and white matter loss (Figure 3) were observed.

##### Somatic Growth

Short stature with short limbs affected all but one individual. Intrauterine growth restriction of the limbs was observed in two of nine patients (22%).

##### Bones

**Figure 4:**
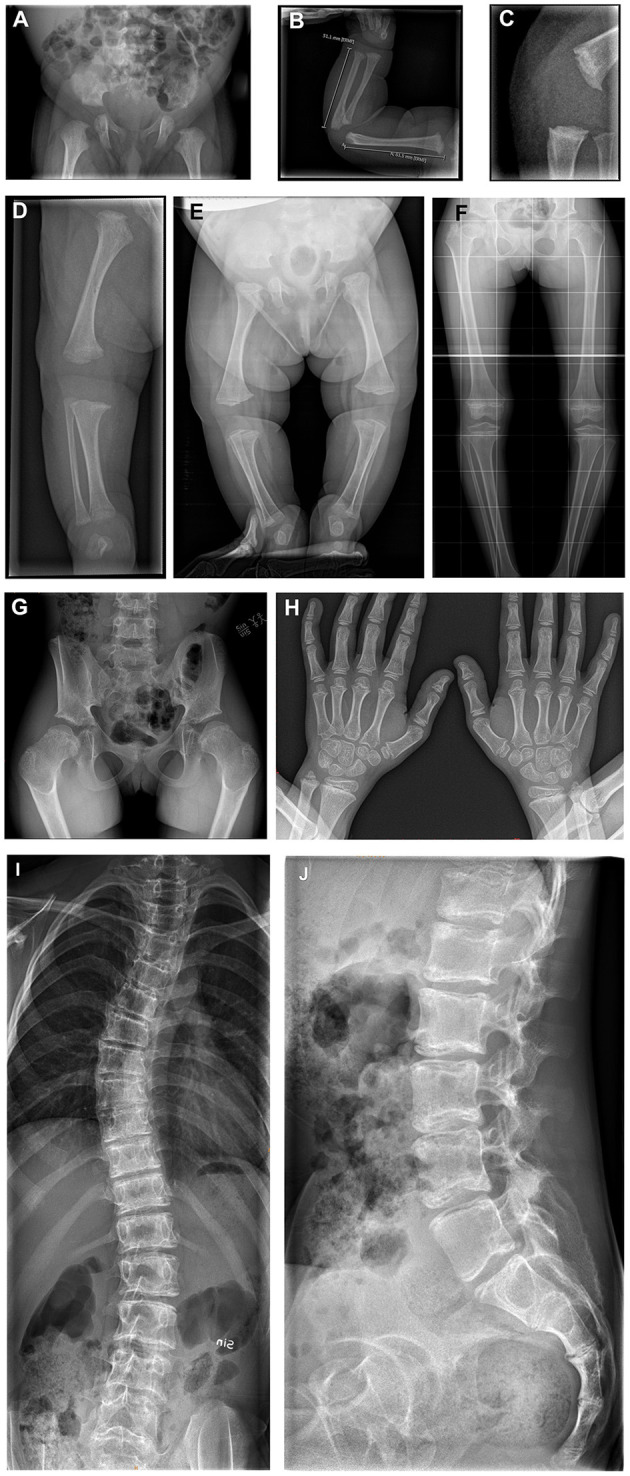
**(A)** X-rays of the skeleton of patients 3, 4, and 6. **(A–D)** Patient 3 at age 4 days; born with multiple congenital abnormalities of the bones. **(A)** The pelvis shows flat acetabula and short femoral necks. **(B)** The right arm demonstrates metaphyseal widening and irregularity. **(C)** The knee in the lateral view demonstrates metaphyseal irregularity in detail. **(D)** The image of the right leg demonstrates irregularly widened metaphyses at the distal femur and proximal and distal tibia, with a dysplastic knee joint and varus deformity. **(E)** Patient 4 at age 4 months; total legs, demonstrating flat acetubular roofs, wide femoral head metaphyses with short femoral necks. There is slight bowing and varus in the knees. The metaphyses around the knee show widening and irregularity. **(F–H)** Patient 6 at age 7 years. **(F,G)** Total legs and pelvic showing small iliac wings, coxa vara with small femoral heads and necks. This is shown in detail in image **(G)**. The metaphyses around the knee demonstrate the typical striated sclerosis. The knee joints are dysplastic, and there is a varus deformity. **(H)** Both hands with irregularly widened and sclerotic metaphyses in the distal radius and ulna. Irregular metaphyses of the phalanges. **(I,J)** Patient 6 at age 12 years; total spine with scoliosis. The vertebral plates seem to have a double layer, with abnormal sclerosis of the plates. Lateral view shows the sclerosing of the vertebral plates from dorsal to ventral, resulting in a double contour.

Short limbs were found on clinical examination in eight of nine patients (89%). Skeletal malformations such as trunk–limb disproportion, coxa vara, and scoliosis had been reported in the first NANS-CDG case series (5). Indeed, skeletal malformations were present in all eight currently reported patients for whom radiographic imaging was available (Figure 4):

*Skull*: frontal bossing (*n* = 2/2; 100%).
*Spine*: spinal deformities (*n* = 5/6; 83%), vertebral sclerosis (*n* = 2/6; 33%), less/irregular calcification of vertebral plates (*n* = 2/6; 33%); abnormal vertebral plates with sclerosis (*n* = 2/6; 33%) and abnormal ossification centers (*n* = 1/6; 17%).
*Pelvis*: short femoral neck (*n* = 5/5; 100%), small femoral head (*n* = 4/5; 80%), small iliac wings (*n* = 3/5; 60%), flat/dysplastic acetabula (*n* = 3/5; 60%), sclerosis of iliac crest (*n* = 1/5; 25%), and luxated hip (*n* = 1/5; 25%).
*Upper extremities*: metaphyseal widening (*n* = 7/8; 88%), metaphyseal sclerosis (*n* = 6/8; 75%), metaphyseal irregularity (*n* = 5/8; 63%), and small epiphyses (*n* = 2/8; 25%).
*Lower extremities*: metaphyseal sclerosis (*n* = 5/6; 83%), metaphyseal widening (*n* = 4/6; 67%), metaphyseal irregularity (*n* = 3/6; 50%), small epiphysis (*n* = 2/6; 33%), dysplastic knee joints (*n* = 1/6; 17%), genu vara (*n* = 1/6; 17%), fibular overgrowth (*n* = 1/6; 17%), and sclerosing knee metaphysis (*n* = 1/6; 17%).

We scoped OMIM database to compare the NANS-CDG phenotype with other CDG-types (Table 2); 49 (38% of the total) CDGs presented with various skeletal anomalies (Table 2), of which 13 of 49 (27%) involve defects in N-glycosylation, 14 of 49 (29%) defects in *O*-glycosylation, 3 of 49 (6%) defects in glycosphingolipid and GPI-anchor glycosylation, and 19 of 49 (39%) defects in multiple glycosylation and other pathways. IDD was present in 40 (82%) of these conditions.

**Table 2:** Overview of skeletal anomalies in CDGs (13, 14)^1^,^2^.

**CDG subtype with skeletal anomalies**	**IDD**	**Protein name**	**Treatable**
ALG1 (OMIM#608540)	Yes	GDP-Man:GlcNAc2-PP-dolichol mannosyltransferase	Unknown
ALG3 (OMIM#601110)	Yes	Dolichyl-P-Man:Man5GlcNAc2-PP-dolichyl mannosyltransferase	Unknown
ALG6 (OMIM#603147)	Yes	Dolichol-P-glucose:Man9GlcNAc2-PP-dolichol glucosyltransferase	Unknown
ALG8 (OMIM#608104)	Yes	Dolichol-P-glucose: Glc1Man9GlcNAc2-PP-dolichol- α1,3-glucosyltransferase	Unknown
ALG9 (OMIM#608776)	Yes	Dolichol-P-mannose:α1,2 mannosyltransferase	Unknown
ALG12 (OMIM#607143)	Yes	Dolichol-P-mannose: Man7GlcNAc2-PP-dolichol mannosyltransferase	Unknown
ALG13 (OMIM#300884)	Yes	UDP-GlcNAc:dolichol pyrophosphate N-acetylglucosamine transferase (cytosolic)	Unknown
ATP6V0A2 (OMIM#278250) ^a^	Yes	The multisubunit vacuolar-type proton pump (H(+)-ATPase or V-ATPase)	Unknown
B3GALT6 (OMIM#271640, #615349)	No	Beta-1,3-galactosyltransferase 6	Unknown
B3GALTL (OMIM#261540)	Yes	*O*-fucose-specific beta-1,3-N-glucosyltransferase	Unknown
B3GAT3 (OMIM#284139)	No	Beta-1,3-glucoronyltransferase 3	Unknown
B4GALNT1 (OMIM#609195)	Yes	Beta-1,4-N-acetylgalactosaminyltransferase 1	Unknown
B4GALT7 (OMIM#130070)	Yes	Golgi UDP-galactose:N-acetylglucosamine β-1,4-galactosyltransferase	Unknown
CHSY1 (OMIM#605282)	Yes	Chondroitin sulfate synthase 1	Unknown
COG1 (OMIM#611209)	Yes	Subunit 1 of the COG complex in Golgi trafficking	Unknown
COG7 (OMIM#608779)	Yes	Subunit 7 of the COG complex in Golgi trafficking	Unknown
COG8 (OMIM#611182)	Yes	Subunit 8 of the COG complex in Golgi trafficking	Unknown
DDOST (OMIM#614507, #602202)	Yes	Subunit DDOST of the OST complex	Unknown
DPAGT1 (OMIM#608093)	Yes	UDP-GlcNAc:dolichol-phosphate-N-Acetylglucosamine-1-phosphotransferase	Unknown
DPM1 (OMIM#608799)	Yes	GDP-Man:Dol-P mannosyltransferase subunit 1	Unknown
DPM2 (OMIM#615042)	No	Dolichol-P-mannose synthase-2	Unknown
EXT1 (OMIM#133700)	No	Exostosin glycosyltransferase 1	Unknown
EXT2 (OMIM#133701)	No	Exostosin glycosyltransferase 2	Unknown
FKTN (OMIM#253800)	Yes	Fukutin	Unknown
FKRP (OMIM#613153)	Yes	Fukutin-related protein	Unknown
GMPPB (OMIM#615350, #615351, #615352)	Yes	GDP-mannose pyrophosphorylase subunit B	Unknown
LFNG (OMIM#609813)	No	*O*-fucose-specific beta-1,3-N-acetylglucosaminyltransferase	Unknown
MPDU1 (OMIM#79323)	Yes	Dol-P-Man utilization 1	Unknown
MGAT2 (OMIM#212066)	Yes	Golgi N-acetyl-glucosaminyltransferase II	Unknown
MOGS (OMIM#606056)	Yes	Endoplasmic reticulum glucosidase I	Unknown
NUS1 (OMIM#617082)	Yes	Subunit of *cis*-prenyltransferase (*cis*-PTase)	Unknown
PGM3 (OMIM#615816, #172100)	Yes	Phosphoglucomutase 3	Unknown
PIGA (OMIM#300868, #300818)	No	Phosphatidylinositol glycan anchor class A protein	Unknown
PIGL (OMIM#280000)	Yes	Phosphatidylinositol glycan anchor biosynthesis class L protein	Unknown
PIGT (OMIM#615398)	Yes	Phosphatidylinositol glycan anchor biosynthesis class T protein	Unknown
PIGY (OMIM#239200)4	Yes	Phosphatidylinositol glycan, class V	Unknown
PMM2 (OMIM#212065) ^a^	Yes	Phosphomannomutase 2	Unknown
POMT1 (OMIM#236670, #613555, #609308)	Yes	*O*-mannosyltransferase 1	Unknown
POMT2 (OMIM#613150, #613156, #613158)	Yes	*O*-mannosyltransferase 2	Unknown
SLC35A3 (OMIM#615553)	Yes	Solute carrier family 35 (udp-N-acetylglucosamine transporter), member 3	Unknown
SLC35C1 (OMIM#266265)	Yes	GDP-fucose transporter	Yes (fucose)
SLC39A8 (OMIM#616721)	Yes	SLC39A8 transporter of divalent cations, including manganese (Mn), zinc (Zn), cadmium (Cd), and iron (Fe)	Yes (dietary galactose)
SRD5A3 (OMIM#612379)	No	Steroid 5 alpha-reductase 3	Unknown
SSR4 (OMIM#300934)	Yes	Signal sequence receptor 4 protein of the TRAP complex	Unknown
TMEM165 (OMIM#614727, #614726)	Yes	TMEM165 (TPARL) protein	Yes (dietary galactose)
TRAPPC11 (OMIM#615356)	Yes	Trafficking protein particle complex, subunit 11	Unknown
VPS13B (OMIM#21655)	Yes	Vacuolar protein sorting 13B, VPS13B	Unknown
XYLT1 (OMIM#615777)	Yes	Xylosyltransferase 1	Unknown
XYLT2 (OMIM#605822)	No	Xylosyltransferase 2	Unknown

a *CDGs in which sialic acid plays a role*.

1 *IEMbase v.2.0.0. Available online at: http://www.iembase.org/index.asp*.

2 *OMIM: Online Mendelian Inheritance in Man. Available online at https://www.omim.org/*.

##### Facial Dysmorphisms

We confirmed the previously reported typical facial gestalt (Figure 2), with a sunken/wide nasal bridge (*n* = 9/9; 100%) and a prominent forehead with frontal bossing (*n* = 6/9; 67%), tent-shaped or prominent mouth (*n* = 6/9; 67%), a prominent upturned nasal tip (*n* = 5/9; 56%), short neck (*n* = 3/9; 33%), synophrys (*n* = 3/9; 33%), teeth abnormalities (*n* = 3/9; 33%), and epicanthus (*n* = 2/9; 22%) (5). Other and new features reported in more than a single patient included hypertelorism (*n* = 5/9; 56%), low-set ears (*n* = 5/9; 56%), tongue protrusion (*n* = 3/9; 33%), large ears (*n* = 2/9; 22%), long eyelashes (*n* = 2/9; 22%), lateral slanted eye(lid)s (*n* = 2/9; 22%), full cheeks (*n* = 2/9, 22%), prominent philtral ridges (*n* = 2/9, 22%), and (mild) macrocephaly (*n* = 2/9; 22%) (Figure 2). Over time, the face may appear coarser.

##### Eye

Abnormal ophthalmological findings were present in six of nine patients (67%). Strabismus was most common, but cone-red dystrophy (retinal disease), amblyopia, myopia, hyperopia, nystagmus (eye movement disorders), and delayed visual development were also observed.

##### Heart

Prompted by the previous reported *nansa* knockdown zebrafish phenotype, showing pericardial edema, an echocardiography was done in patient 1, which revealed a dilated aortic root and abdominal aorta, hitherto asymptomatic (5). No other cardiac abnormalities were reported.

#### Newly Observed Clinical Features

##### Hearing Problems

Hearing problems were present in two of nine patients (22%): conductive hearing loss (due to middle ear fluid) and perceptive hearing loss with an abnormal brainstem evoked response audiometry (BERA).

##### Gastrointestinal and Nutrition

**Figure 5:**
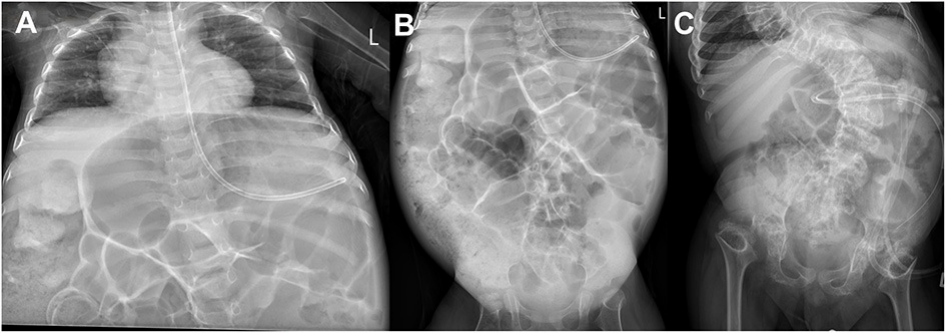
Abdominal X-rays of patients 1 and 3. **(A,B)** Patient 3 at age 2 years; severe dilatation of the stomach and bowel loops and fecal impaction especially in the right colon. **(C)** Patient 1 at age 8 years; a similar stomach and bowel loop dilatation.

Four of nine patients (44%) developed feeding difficulties and failure to thrive, requiring tube feeding temporarily or permanently. Severe constipation with abdominal distention (Figure 5) requiring laxatives or even daily enemas with negative impact on quality of life were present in five of nine patients (56%). For two patients, histopathological studies were performed to rule out Hirschsprung disease.

##### Urinary Tract

Three of nine patients (33%) suffered urinary tract abnormalities: neurogenic bladder, urethral stenosis with hydronephrosis, and bladder-emptying disorder. No congenital malformations of the kidneys were reported.

##### Infections

A common feature of NANS-CDG was a predilection toward recurrent infections, including upper and lower respiratory tract, ears, and urinary tract. In one patient, two respiratory infections that did not respond well to treatment resulted in a prolonged hospital stay. However, progression to severe infections or sepsis was not reported.

##### Laboratory Findings

Thrombocytopenia was observed in five of eight patients (63%), in whom thrombocyte counts were measured, varying from 29 × 10^9^/L to 192 × 10^9^/L. In one case, platelet transfusions were needed approximately every 1–2 weeks; the other four did not suffer increased bleeding tendency. Mild anemia was detected in two of eight patients (25%), in whom red blood cell counts were measured, normocytic in one patient [hemoglobin (Hb) 5.4 mmol/L; reference range = 6.5–8.7 mmol/L], and microcytic in the other patient (Hb 5.7 mmol/L; reference range = 7.5–9.9 mmol/L), both likely due to dietary restriction.

Low levels of LDL cholesterol were detected in four of seven patients (57%) in whom these were measured: in three patients during childhood (varying from 0.78 to 1.49 mmol/L; reference range = 1.75–3.25 mmol/L for patients 2 and 3; reference range = 1.61–3.37 mmol/L for patient 7) and in one patient in adulthood (1.52 mmol/L; reference range = 1.76–4.09 mmol/L).

In three of eight patients (38%), in whom liver function tests were performed, mildly elevated alkaline phosphatase was reported (varying from 314 to 462 U/L; reference <155 U/L for patients 2 and 3; reference range = 124–341 U/L for patient 4). In one patient (13%), a decreased alkaline phosphatase was reported (82 U/L; reference range = 110–440 U/L), likely due to dietary restriction. Lactate was elevated in two of five patients (40%), in whom lactate was measured (varying from 4.2 to 7.0 mmol/L; reference range = 0.8–2.1 mmol/L) and normalized thereafter (Table 3).

**Table 3:** Overview of disease features in 17 reported NANS-CDG cases.

**Reported symptoms**	**Frequency^**a**^**	**%**
IDD	17	100
Abnormal muscle tone	17	100
Short stature	16	94.1
Facial dysmorphisms	15	88.2
Skeletal dysplasia^b^	14	82.3
Short limbs^c^	13	76.5
Ocular abnormalities	11	64.7
Brain septum pellucidum abnormalities	8	47.1
Joint hypermobility	7	41.2
Recurrent infections	6	35.3
Gastrointestinal dysfunction	6	35.3
Thrombocytopenia	5	29.4
Seizure history	5	29.4
Hypo-LDL cholesterolemia	4	23.5

a *Frequency of all symptoms based on current reported patients and cases described by van Karnebeek et al. (5), according to findings documented in the published manuscript and supplemented clinical patient reports*.

b *Skeletal X-rays were not available in three patients [patient 8 in current report and patients 6 and 7 in the 2016 report (5)]*.

c *Measured on clinical examination*.

### Analysis of the Genotype–Phenotype Correlation

Three patients (patients 1, 2, and 3) harboring the same pathogenic variants, c.709C>T and c.562T>C, were considered to be at the severe end of the phenotypic spectrum. Their MRI scans revealed extensive neurological anomalies, and radiographic imaging showed anomalies in all parts of the skeletal system. Moreover, ophthalmic involvement, abdominal distention, and thrombocytopenia were reported in all three patients. Shared variants were not found in any of the other patients.

### Analysis of the Biochemical–Clinical Correlation

**Figure 6:**
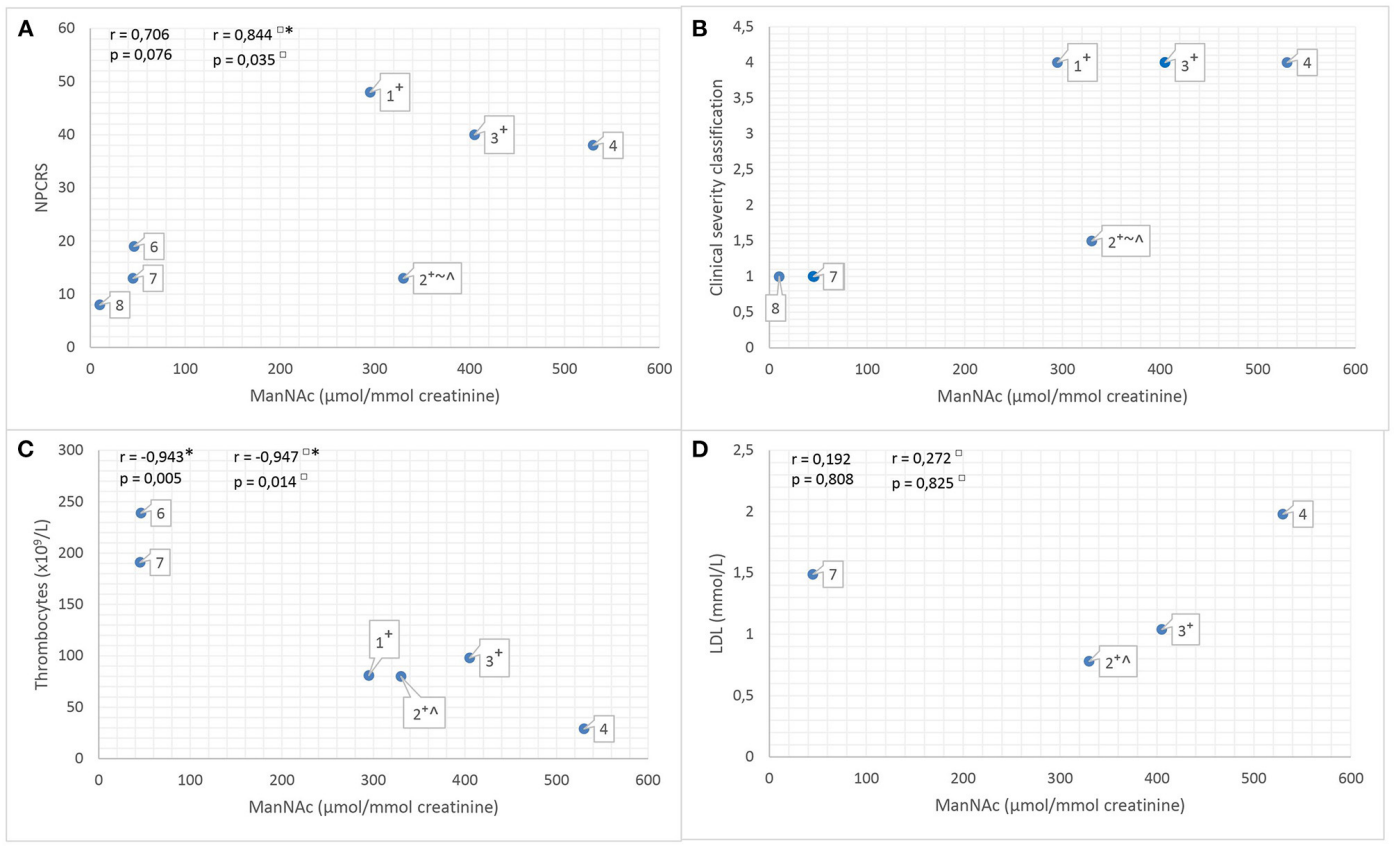
**(A)** ManNAc excretion levels in urine (μmol/mmol creatinine, measured by ^1^H NMR spectroscopy, reference value not detected) vs. the Nijmegen Pediatric CDG Rating Scale (NPCRS) for currently reported patients in whom a ManNAc excretion level was determined. **(B)** ManNAc excretion levels in urine (μmol/mmol creatinine, measured by ^1^H NMR spectroscopy, reference value not detected) vs. the clinical severity classification for currently reported patients in whom a ManNAc excretion level was determined. **(C)** ManNAc excretion levels in urine (μmol/mmol creatinine, measured by ^1^H NMR spectroscopy, reference value not detected) vs. thrombocyte count for currently reported patients in whom a ManNAc excretion level and thrombocyte counts were measured. If the thrombocyte counts were measured several times, we used the lowest value. **(D)** ManNAc excretion levels in urine (μmol/mmol creatinine, measured by ^1^H NMR spectroscopy, reference value not detected) vs. LDL level for currently reported patients in whom a MaNAc excretion level and LDL were measured. If the LDL level was measured several times, we used the lowest value. **(A–D)** Label numbers indicate the patients. □, analyses excluding patient 2; *, significant at the 0.05 level; †, patients harbor the same mutation [c.709C>T p.(Arg237Cys); c.562T>C p.(Tyr188His)]; ~, in patient 2 (aged 3 months) the NPCRS score and clinical severity classification were low compared to his ManNAc excretion level. Important developmental milestones are not relevant at this young age, explaining why the clinical severity classification is lower than expected on the basis of ManNAc excretion level; ∧, patient is treated with prenatal and postnatal experimental sialic acid; CDG, congenital disorder of glycosylation; ManNAc, *N*-acetylmannosamine; ^1^H NMR, quantitative proton nuclear magnetic resonance; NPCRS, Nijmegen Pediatric CDG Rating Scale; r, Pearson linear correlation coefficient.

To assess the disease severity, we tested the use of the NPCRS model and the clinical severity classification. We calculated Pearson linear correlation coefficient *r* between the NPCRS score [range = 3 (mild) to 48 (profound)], LDL levels, thrombocyte counts, and ManNAc excretion in urine (reference value not detected; Figures 6A,C,D). The positive correlation between ManNAc excretion and NPCRS score (*r* = 0.706; *p* = 0.076; Figure 6A) and between ManNAc excretion and LDL levels (*r* = 0.192; *p* = 0.808; Figure 6D) was not significant. We found a significant negative correlation between ManNAc and thrombocyte counts (*r* = −0.943; *p* = 0.005; Figure 6C). Upon visual examination of the data in Figure 6B, a positive correlation between ManNAc excretion and the clinical severity classification was revealed. For two patients, ManNAc excretion could not be determined because of unavailability of a urine sample. There was one outlier: in patient 2 (the youngest patient, prenatally treated with sialic acid), a high ManNAc excretion level (330 μmol/mmol creatinine) was reported, but he had a low NPCRS score [(15); Figure 6A]. Analyses excluding patient 2 revealed a positive significant correlation between ManNAc excretion and NPCRS score (*r* = 0.844; *p* = 0.035; Figure 6A) and a significant negative correlation between ManNAc excretion and thrombocyte counts (*r* = −0.947; *p* = 0.014; Figure 6C). All these analyses suggest a positive correlation between biochemical and clinical phenotype: i.e., the higher the ManNAc excretion, the more severe the phenotype.

### Experimental Sialic Acid Supplementation

The neurodegeneration observed in patient 3 prompted us to pursue the prenatal experimental therapy with sialic acid in patient 2. The prenatal sialic acid treatment (dose of 4,000 mg/m^2^ per day in four doses) initiated during the third trimester of pregnancy was well tolerated by mother after initial polyuria, as well as postnatally by patient 2. A separate publication will provide details on the experimental sialic acid therapy for this and three other patients in this report, who started at age 2 (patient 3), 16 (patient 7), and 28 years (patient 9).

## Discussion

CDGs belong to an expanding and heterogeneous group of inherited metabolic disorders, which pose challenges regarding diagnosis and patient management. A precision medicine approach with rapid diagnostics and improved therapeutic interventions is much needed to tackle these challenges and improve patient outcome. Here we present such advances for NANS-CDG patients. Our study of nine affected individuals confirmed the earlier reported clinical hallmarks of IDD with delayed achievement of early developmental milestones, neurologic impairment, recognizable facial gestalt with coarsening over time, and short stature with short limbs. Prenatal diagnosis of one patient was suspected based on these clinical features as observed by ultrasound and confirmed by molecular and biochemical testing. Detailed phenotyping of our cohort revealed new features for NANS-CDG: brain septum pellucidum abnormalities with cortical atrophy and ventricular dilatation, severe abdominal distention, and gastrointestinal dysfunction (intermittent), thrombocytopenia, and low levels of LDL cholesterol. Even though the clinical spectrum of NANS-CDG is wide, this CDG subtype is specifically recognizable by the combination of short stature and short limbs, facial dysmorphisms, skeletal dysplasia, IDD, and neuroimaging findings. However, mild cases may well be missed especially at a young age. For patient 8, the urinary excretion of ManNAc equaled the lower detection limit of the assay (10 μmol/mmol creatinine), but this was still increased compared to controls in which no ManNAc can be detected. Despite this just marginally increased ManNAc excretion, the variants in *NANS* and his phenotype, albeit mild, were very suggestive of the diagnosis NANS-CDG.

To aid the diagnostic process, ManNAc may serve as a specific biomarker for NANS-CDG both in plasma and urine, and our study showed that ManNAc excretion levels significantly correlate with disease severity, i.e., NPCRS score and thrombocyte count. It must be noted that the CDG rating scale NPCRS does not include congenital brain abnormalities and skeletal dysplasia, which makes it less applicable for NANS-CDG. Therefore, we graded the clinical severity classification and examined specific phenotypic features such as skeletal anomalies. Nevertheless, use of ManNAc as a solid biomarker must still be confirmed in larger patient cohorts, also evaluating the influence of age on ManNAc levels. In addition, the three patients (patients 1, 2, and 3) with the highest ManNAc excretion levels had an identical genotype and a severe phenotype with additional clinical features aside from the hallmark features. Speculatively, specific genotypes may relate to ManNAc concentrations. The additional features in these patients, including congenital brain abnormalities, as well as neurodegenerative features both clinically and on MRI scan, ocular abnormalities, abdominal distension, and thrombocytopenia, motivated us to intervene via an experimental treatment with oral sialic acid. We started prenatal treatment during the third trimester in the youngest of the three patients (patient 2) and upon good tolerability and potential beneficial effects, subsequently in patients 3 at age 2 years, 7 at age 16 years, and 9 at age 28 years.

The therapeutic rationale was based on the *nansa* knockdown zebrafish studies, which demonstrated partial rescue (50%) of the skeletal phenotype with early oral sialic acid supplementation (5). As expected, our prenatally treated patient was born with congenital brain abnormalities and skeletal dysplasia, as they have their origins earlier in pregnancy, prior to treatment. Interestingly, at age 7 months, this boy's neurologic features were milder than the other two genotypically identical patients. He makes more progress in development; he attained the ability to roll at 5 months and to sit at 8 months. Longer follow-up of clinical well-being and disease course, somatic growth, and neurodevelopment is required. The results of sialic supplementation in these patients will be published after a minimum of 12 months' follow-up.

To establish genotype–phenotype correlation, the number of NANS-CDG patients is too limited. The c.709C>T [p.(Arg237Cys)] variant that was identified heterozygous in four patients and homozygous in one patient presented biochemically with high ManNAc excretion levels and clinically with severe skeletal dysplasia, severe growth restriction, brain abnormalities, and thrombocytopenia. Information of this variant may guide genetic counseling in the future. Larger cohort studies with more patients, diverse genotypes, detailed phenotyping, and longer follow-up are needed for genotype–phenotype predications.

All patients showed neurologic dysfunction, ranging from mild to very severe. Psychomotor development is usually delayed in NANS-CDG patients and already evident in the first months of life. Sialic acid is present on glycoproteins and glycolipids and is highly expressed in the human brain (2). Two studies reporting on patients with psychomotor delay, hypotonia, and dysmorphic features found variants localized in *SLC35A1* (OMIM#603585) encoding the Golgi CMP–sialic acid transporter (15, 16). Genetic deficiency of sialyltransferase *ST3GAL3* (OMIM#611090) (17–22) and *ST3GAL5* (OMIM#609056) (23, 24), two enzymes that add sialic acid residues to glycoproteins and glycolipids, leads to infantile epilepsy and developmental delay. These observations underline the relationship between sialylation and brain functions and suggest that sialylation of proteins and lipids are necessary for brain development.

A remarkable finding on brain MRI is the abnormal formation of the septum pellucidum, which could be secondary to low levels of LDL cholesterol, as structural anomalies involving the midline and paramidline are frequently reported in neurodevelopmental disorders caused by errors of cholesterol metabolism, such as Smith–Lemli–Opitz syndrome (OMIM#270400) (25). Our finding of a 75% rate of persistent cavum septum vergae/pellucidum is clearly different from the rate of an enlarged (>6 mm) cavum septum pellucidum in healthy children (4.6%) (26). A persistent cavum septum pellucidum is also seen in MAGT1-CDG (OMIM#301031) and B3GALTL-CDG (OMIM#261540) patients (27, 28) and suggests an association between midline defects of the brain, abnormal glycosylation, and low levels of (LDL) cholesterol. Nevertheless, the clinical significance is unclear, as a cavum septum pellucidum is not generally thought to be symptomatic, and no relationship between intelligence, emotional, and behavioral functioning can be found (25, 26). Other neuroimaging findings, such as congenital structural abnormalities, cortical atrophy, and ventricular dilation, were found and may be caused by yet unrecognized functions of ManNAc, sialic acid, and/or other abnormal metabolites. On the other hand, sialic acid is important for brain formation, and some features could well-result from sialylated glycoprotein deficiencies. Further research in cell and animal models as well as larger cohort studies might yield further insights in the molecular mechanisms underlying neurodegeneration in NANS-CDG.

Low LDL levels have not been previously reported in NANS-CDG patients. This may be due to the lack of a consensus on how to define decreased LDL cholesterol levels, as focus is usually on high LDL levels, as these are commonly considered as an established risk factor for cardiovascular disease. Nevertheless, too low LDL levels might interfere with normal cellular functions, especially in organs that have higher lipid demand, such as the brain. LDL cholesterol is therefore essential for normal (fetal) growth and development (29). Hypocholesterolemia in CDG is usually attributed to an increased receptor-mediated cholesterol uptake through increased LDL receptor expression, leading to low LDL plasma levels (30). Hypercholesterolemia is also seen in some CDG type II subtypes, as an exceptional feature (31–33). These studies may indicate a molecular origin of abnormal LDL values in NANS-CDG and other CDG subtypes.

We observed thrombocytopenia as a new NANS-CDG feature. This is a common tissue-specific feature in diseases associated with reduced sialylation, seen in patients with genetic variants in the kinase domain of *GNE* (34), in *SLC35A1* (35), and in septic patients (36). A recent study on the mechanism of thrombocytopenia in SLC35A1-CDG (OMIM#603585) proposed that sialylation is the major capping glycan structure on megakaryocytes and platelet membrane glycoproteins; in mice with sialylation defects, impaired megakaryocyte maturation and excessive platelet clearance in the liver were found (35). These findings suggest a strong link between sialylation and platelet homeostasis. However, the degree of thrombocytopenia varied in recent and previously described cases (15, 37–40), which is not yet fully understood. Future measurements of serum thrombopoietin levels in NANS-CDG patients may help to unravel the complete pathophysiology.

Elevated alkaline phosphatase, as measured in NANS-CDG patients, is indicative of a high bone turnover, which seems the likely etiology in NANS-CDG (41). Measurements of alkaline phosphatase should be considered in patients with NANS-CDG, as it may serve as a biomarker for diagnosis, prognostication, and treatment. Further research is needed to gain more insight into its values and its role in pathophysiology.

Skeletal dysplasia with short stature, metaphyseal widening, and spine deformations was reported in nearly all cases and in previously reported NANS-CDG patients (5). The defects are the same as in this previous report but of varying severity. The overview of skeletal anomalies in CDGs may help narrow down the differential diagnoses. Fourteen CDGs in this overview are N-glycosylation disorders, which can be diagnosed by isoelectric focusing of serum transferrin. If this test is negative, the clinician knows which CDGs should be pursued, one of which is NANS-CDG. Also, this overview increases awareness among clinicians that CDGs, not only storage disorders, can present with skeletal abnormalities.

Skeletal defects are broadly observed in other CDG subtypes, and there appears to be a diverse spectrum, with most CDG types having multiple skeletal manifestations, such as rhizomelia, contractures, short stature, small hands and feet, and camptodactyly (13). van Karnebeek et al. stated that several key factors in cartilage and bone growth and development, such as chondroitin sulfate proteoglycans, bone sialoprotein, and osteopontin, are sialylated (5). In this line, Xu et al. demonstrated that expressions of bone sialoprotein (BSP), osteoprotegerin (OPG), and vitamin D receptor were significantly decreased when sialic acid expression decreased on the cell surface, affecting bone mineralization (42). Although sialic acid plays a role in only two of the CDGs that present with skeletal anomalies, the hyposialyation state in NANS-CDG may significantly interfere with normal bone growth. A separate study (i.e., systematic review) is needed to give a complete overview of the various skeletal anomalies in CDGs.

We found gastrointestinal dysfunction and abdominal distention as new characteristic features of NANS-CDG. The treatment modalities of patients, i.e., tube feeding and iron and herbal supplements, can cause and exacerbate abdominal distension. However, the abdominal distention was already present in the patients before starting these treatments. Also, the gastrointestinal dysfunction and abdominal distention were so severe that Hirschsprung disease was suspected in two patients. Future research is needed to unravel the mechanism underlying gastrointestinal dysfunction and abdominal distention in NANS-CDG patients.

Limitations of this study include the retrospective design with varying availability of clinical data. Data of Dutch patients were obtained through the patients' electronic medical records. However, access to patient records of non-Dutch patients was limited to their physicians. Additionally, we cannot rule out any variability in the interpretation of the clinical features per patient, described by different clinicians from various hospitals. Second, in two patients, determination of ManNAc excretion in urine was not yet possible at the time of inclusion because of unavailability of urine samples, lessening the strength of biochemical–clinical correlations. Third, reassessment of brain imaging of the Dutch, Hungarian, and German patients was performed by a neuroradiologist of the RUMC, whereas only written neuroimaging reports were available for the other patients. Therefore, no reassessment could take place. Lastly, we provided only short-term follow-up of one prenatally treated individual here. Since then, three other patients have been started on experimental therapy with oral sialic supplementation. The results of this experimental treatment will be reported in a separate study.

In summary, our study confirmed previous data and adds some new hallmark features to the clinical spectrum of NANS-CDG. In addition, we found ManNAc to be a reliable biomarker for biochemical diagnosis and severity scoring. More patients are needed to further explore genotype–phenotype correlations. Future research should focus on the natural history of NANS-CDG and on expanding disease biomarkers to better understand the progression of the disease. Similarly, more research on sialic acid metabolism is required to unravel the complete pathophysiology, including the role of cholesterol in congenital and progressive brain abnormalities. Computer-aided facial phenotyping (DeepGestalt), which can be highly informative to clinicians for syndrome identification, is planned, and these results will be included in a next manuscript. Early recognition of symptoms and accurate genetic and metabolic counseling are fundamental to provide personalized care for NANS-CDG patients. Therapy should focus on managing cognitive impairment, gastrointestinal dysfunction, thrombocytopenia, and seizures. Clinical trials on sialic acid as a potential therapeutic option for improving neurologic, systemic, and growth outcomes in NANS-CDG are ongoing. Results of the experimental trial will be used to improve disease course and elaborate treatment strategies.

## Data Availability Statement

The original contributions presented in the study are included in the article/Supplementary Material, further inquiries can be directed to the corresponding author/s.

## Ethics Statement

Ethical review and approval was not required for the study on human participants in accordance with the local legislation and institutional requirements. Written informed consent to participate in this study was provided by the participants' legal guardian/next of kin. Written informed consent was obtained from the minor(s)' legal guardian/next of kin for the publication of any potentially identifiable images or data included in this article.

## Author Contributions

BH: participated in data collection, data interpretation, and manuscript writing. AR: participated in data collection, data interpretation, and report writing. MP: performed laboratory analysis, participated in data collection, and data interpretation. WK, SF, CH, BJ, AL, KL, EØ, GP, RS, MS, KS, ÓT, UU, and MW: participated in phenotyping and data collection. MO and LB: participated in data collection and data interpretation. MB: participated in data interpretation. UE and LK: participated in laboratory analysis, participated in data collection, and data interpretation. PE: participated in data collection. KC: supervised laboratory analysis and data interpretation. DL: participated in study design, data interpretation, and manuscript writing. CK: supervised study design, data collection, data interpretation, and manuscript writing. All authors provided critical feedback, read, and approved the final manuscript.

## Conflict of Interest

BJ and KS were employed by the company Decode Genetics/Amgen, Inc. The remaining authors declare that the research was conducted in the absence of any commercial or financial relationships that could be construed as a potential conflict of interest.

## Abbreviations

BERA: brainstem evoked response audiometry
BSP: bone sialoprotein
CADD: combined annotation-dependent depletion
CDG: congenital disorder of glycosylation
IDD: intellectual developmental disorder
LDL: low-density lipoproteins
ManNAc: *N*-acetylmannosamine
NANS: *N*-acetyl-d-neuraminic acid synthase
NGMS: next-generation metabolomic screening
^1^H NMR: quantitative proton nuclear magnetic resonance
NPCRS: Nijmegen Pediatric CDG Rating Scale
OPG: osteoprotegerin
RCDG: Radboudumc Center of expertise on Glycosylation Disorders
RUMC: Radboud University Medical Center
SLO: Smith-Lemli-Opitz syndrome
WES: whole-exome sequencing.
