# Supplementary material for: NANS-CDG: Delineation of the Genetic, Biochemical, and Clinical Spectrum
Source: Front Neurol. 2021 Jun 7;12:668640. doi: 10.3389/fneur.2021.668640 (PMC8215539; doi:10.3389/fneur.2021.668640)
Supplement: Supplementary file 1 [file Data_Sheet_1.docx]

Supplementary Material

**File 1: clinical patient reports**

**Patient 1**

**This 8 year-old male patient is previously reported by Van Karnebeek, C et al. (patient 9 in this previous report (1)). Family history included oculocutaneous albinism on maternal side, no** consanguinity**. He was born at 41 weeks and 3 days of gestation to parents of Dutch descent after an uneventful pregnancy via vaginal delivery. Birth weight was 2865 g (P4), Apgar scores unknown. Short limbs and neonatal jaundice, requiring a few days of phototherapy, were present.**

**At 3 weeks of age, he was admitted to the hospital because of abdominal distention and failure to thrive. An allergy to cow milk was diagnosed. His growth improved little after formula was changed, however a G-tube was required. On follow-up at 3 months he showed short stature with short limbs (impression of skeletal dysplasia), hypotonia, global developmental delay and facial dysmorphisms (prominent forehead, sunken nasal bridge with upturned nasal tip, tented shaped mouth, mild macrocephaly, low set posteriorly rotated large ears, full cheeks, thick eyebrows, long eyelashes). A brain MRI at 4 months showed hydrocephalus. During the first months of life he developed epileptic encephalopathy with hypsarrythmia. Global development was profoundly delayed: unaided sitting, unaided walking and complete head control (able to lift head when lying) were never achieved, he was non-verbal and minimally reactive.**

**Constipation with intermittent abdominal pain and distention (Figure 5), partial bowel obstruction, cortical visual impairment, severe skeletal dysplasia (e.g. frontal bossing, scoliosis and kyphosis, and metaphyseal widening of the upper limbs), laryngomalacia post-laser surgery, recurrent infections (aspiration pneumonias, urinary tract infections), a neurogenic bladder, obstructive sleep apnea, conductive hearing loss due to middle ear fluid and signs of dysautonomia were also reported. Cardiac evaluation revealed a dilated aortic root and abdominal aorta. Neuroradiological follow-up revealed ventriculomegaly, cerebral atrophy with white matter reduction, persistent cavum vergae, dysmorphic basal nuclei, hypoplasia of the corpus callosum and splenium, aplasia of the rostrum and asymmetry of the cerebellum.**

**Diagnosis was made at age 3 years based on** Whole Exome Sequencing **(WES) via the TIDEX research study and revealed compound heterozygous variants of *NANS*. At this same age,** ManNAc excretion in urine was 295 µmol/mmol creatinine (reference value ‘not detected’)**. At last clinical follow-up (age 8 years), his height was 91 cm (<P0,01), weight 19,8 kg (<P0,5), head circumference not measured, G-tube in situ. Medications include piracetam, esomeprazole, laxatives, CBD oil, elemental iron, calcium/magnesium, midazolam, salbutamol, probiotics and multiple herbal supplements. Laboratory findings revealed leukopenia (4,2x10^9^/L; reference range 3,9-10,2x10^9^/L, recovered), thrombocytopenia (81-113**x10**^9^/**L; reference range 180-440x10^9^/L) and low alkaline phosphatase (82 U/L; reference range 110-440 U/L) at 8 years. **Electrolytes, TSH, k**idney and other liver function tests were normal, as were red blood cell counts. He is home schooled through a distance education curriculum; he participates in music therapy. Nurses participate in his care via day and night shifts. He communicates primarily with facial expressions and started to communicating with a communication device and eye gaze technology.

**Patient 2**

A 3-month old boy, part of a Dutch family, was born as the second child to non-consanguineous parents, after NANS-deficiency was prenatally diagnosed due to abnormal growth velocity of both brain and skeletal tissue and other remarkable findings (short limbs, polyhydramnios, dyspmorphic features). On prenatal 3D ultrasound at 34 weeks and 2 days of gestation facial dysmorphisms were recognizable (**Figure 2**). Pregnancy was complicated by a vanishing twin at 8 weeks of gestation. No other issues concerning family history were noted. At 28 weeks of gestation, brain MRI showed hypoplastic cerebellum and subependymal pseudocysts and a deflection in the growth of the head (P<3 at 32 weeks and 3 days of gestation) and femur (P<3 at 28 weeks and 6 days of gestation) was reported. Genetic counselling revealed bi-allelic *NANS* variants reported previously in case 9 in 2016 (1), after which maternal sialic acid administration was started at 34 weeks of gestation. He was born at term; birth weight was 3000 g (P7), Apgar scores 6; 8; 9. Metabolic compensated respiratory acidosis, thrombocytopenia, hypotonia, feeding problems (requiring tube feeding), yellow appearance (bilirubin level just below limit requiring phototherapy) and undeveloped limbs were noted in the neonatal period. Remarkable facial features were sunken nasal bridge, hypertelorism, posteriorly rotated ears, short neck, lateral slanted eyes and tongue protrusion. In his first weeks of life, radiographic examination of his skeleton showed metaphyseal widening of the upper and lower limbs with sclerosis and irregularity. Brain MRI at age 2 days showed a fetal gyral pattern with simplified sulcation, thin corpus callosum with hypoplastic splenium, widened ventricles and cisternae, subependymal pseudocysts and a cavum septum pellucium (**Figure 3**).

After discharge, the feeding tube was removed. The patients’ mother continued with sialic acid to treat him via breastfeeding. At age 12 days, maternal treatment was stopped, and sialic acid was added to the regular infant formula. After 21 days, the dosage was increased to 750 mg/day (which equals 4000 mg/m^2^, in 4 doses). Clinical follow-up after 1-3 months demonstrated axial hypotonia with full head lag, little eye contact, positional preference for right and abdominal distention; vision, hearing, muscle strength and cardiac function were normal. At 3 months of age, his height was 56 cm (<P0,4), weight 5435 g (P8) and head circumference 41 cm (P85). Beside the sialic acid, no other medication was prescribed. Blood chemistry workups yielded thrombocytopenia (varying between 80x10^9^mmol/L (reference range 150-450x10^9^/L) and 189x10^9^mmol/L (reference range 210-650x10^9^/L)), elevated alkaline phosphatase (374-462 U/L; reference <155 U/L) and low LDL levels (0,78-1,1 mmol/L; reference range 1,75-3,25 mmol/L). White and red blood cell counts, lactate, electrolytes, kidney function and other liver functions tests were normal. Metabolic testing confirmed the biochemical signature with elevated ManNAc excretion in urine of 330 µmol/mmol creatinine at 2 months of age (reference value ‘not detected’).

**Patient 3**

This boy, 2,5 years old, is the first child of healthy, Dutch parents. There is no family history or consanguinity of note. At gestational age of 28 weeks, deflection in growth of all limbs (femur length <P0,1) was noted as well as brain ultrasound abnormalities: small cerebellum but with normal configuration, small cavum septum pellucidum, mild ventriculomegaly and suspected syntelencephaly (not present on post-partum MRI of the brain). He was born at term via a spontaneous vaginal delivery with a birth weight of 3228 g (P23), Apgar scores 3; 8; 10, axial hypotonia, hyperlaxity and short limbs. Facial dysmorphisms included frontal bossing, sunken nasal bridge with upturned nasal tip, short neck, bulbous eyes with slight hypertelorism, tent shaped mouth with thin upper lip, frequent tongue protrusion and low-set ear position. He required short-term continuous positive airway pressure (CPAP) postnatally, as well as 3 days of phototherapy for neonatal jaundice, and a nasogastric tube because of feeding problems due to dysfunctional swallowing. Cardiac ultrasound was unremarkable. Brain MRI at 4 days postnatally showed increased ventriculomegaly, absence of septum pellucidum, limited volume of the corpus callosum and periventricular white matter, and the suggestion of a cortical malformation of the left temporo-parietal region (**Figure 3**). Radiographic images at age 4 days showed frontal bossing, metaphyseal widening of the upper and lower limbs with sclerosis and irregularity, small iliac wings, irregular acetabula, small and short femoral neck and abnormal vertebra (**Figure 4**).

Global developmental delay was present with a delay in all milestones: severe head lag with minimal head control at 2 years of age with a preference for right side, rolling from side to side at 18 months, grasping between 18 and 20 months and not yet able to sit. There is no speech or language development, and limited but increasing social interaction. Developmental testing, according to the Bayley Scale of Infant and Toddler Development, revealed a score of 49 at age 17 months (cut-off score for neurodevelopmental delay: <80). Ophthalmology testing showed delayed visual development. Auditory function was normal.

The diagnosis NANS-CDG was established at age 6 months of age, via WES which revealed bi-allelic *NANS* variants reported previously in case 9 in 2016 (1). Subsequent metabolic testing confirmed the biochemical signature with elevated ManNAc excretion in urine of 405 µmol/mmol creatinine (reference value ‘not reported’).

He suffered severe gastro-intestinal problems with excessive mucus production and vomiting after feeding and abdominal distention **(Figure 5)** requiring bowel enema. During first two years of life, he was admitted to the hospital once due to a respiratory infection with low intake (age 6 months), once due to impending dehydration (age 10 months) and once for colon cleansing because of severe obstipation (age 23 months). Because of suspected Mb. Hirschsprung at 2 years of age, microscopic examination of the gut was performed, however did not confirm Mb. Hirschsprung.

Radiographic images at age 28 months showed increasing kyphosis and a dysplastic left hip with luxation. EEG was normal.

Brain MRI was repeated at age 2 years, showing progressive cerebral atrophy, consisting of supratentorial white matter loss including the optic chiasm and corpus callosum, atrophy of the basal ganglia and enlargement of the lateral ventricles (mostly ex vacuo, perhaps combined with mild CSF flow obstruction due to a relative aqueduct stenosis) (**Figure 3**).

At age 26 months the patient started with sialic acid experimental therapy. Upon examination at 26 months, his height was 76,2 cm (P0,01), weight 9,426 kg (P0,01) and head circumference (48,3 cm) remained on -1 SD despite evident cerebral atrophy on MRI, which raised suspicion of mildly increased intracranial pressure related to the ventriculomegaly and narrow aqueduct. However, there was no optic nerve edema and no transependymal migration of CSF.

Overall, he appeared a happy child despite the gastro-intestinal problems, requiring enemas twice a day, no other medication. Neurologically he showed some progress with minimal head control, onset of grasping, reduced right-sided preference, improved social interactions with better eye contact and following, smiling and cooing. Axial hypotonia, hypertonia of limbs, dystonia and a tremor were noted. Laboratory studies throughout the first two years of life marked low LDL-levels (1,04-1,16 mmol/L; reference range 1,75-3,25 mmol/L ), mild thrombocytopenia (98x10^9^/L-192x10^9^/L; reference range 210-430x10^9^/L), and elevated lactate (7,0 mmol/L; reference 0,8-2,1 mmol/L). Liver function showed elevated alkaline phosphatase (314 U/L; reference <155 U/L), ALAT and ASAT were in the normal range. Kidney function, electrolytes, thyroid function and cortisol were normal, as were white and red blood cell counts.

**Patient 4**

A 10-month old girl, daughter of non-consanguineous Hungarian parents and no family history of note, was born at 37 weeks of gestation after un uncomplicated pregnancy. Birth weight was 2190 g (P<2.5), Apgar scores 9; 10; 10, hydrocephalus, skeletal dysplasia (a phenotype of short limbs) and mild neonatal jaundice (no phototherapy required) were reported. Developmental milestones were severely delayed in the first year; limb movements were limited, head control and sitting were not attained at age 10 months. She turned from side to back, lower extremities seem stronger, but shoulder girdle was very weak. Social interaction is limited, however, she looks at her mother during feeding. Because of suspected peritonitis and Mb. Hirschsprung at 3 months of age, soave operation and microscopic examination of the gut was performed, however did not confirm Mb. Hirschsprung. A cerebral MRI at age 8 months showed enlarged lateral ventricles and third ventricle, aquaduct stenosis, absent splenium of the corpus callosum and cerebral atrophy. During the first months of life she developed West syndrome with intractable seizures, strabismus, skeletal dysplasia with metahpyseal widening of the upper and lower limbs with sclerosis and irregularity; genu vara (**Figure 4**), urethral stenosis with hydronephrosis (left kidney indicated surgery at last follow-up), constipation with abdominal distention and recurrent upper respiratory tract infections which resulted in prolonged hospital stays. Cardiac and vision evaluations were normal. Brainstem Evoked Response Audiometry testing was abnormal on the left side. Diagnosis was made at age 10 months based on targeted next-generation sequencing and revealed compound heterozygous *NANS* variants. ManNAc excretion in urine was 530 µmol/mmol creatinine (reference value ‘not detected’) at age 10 months. On examination at 10 months, her height was 57 (<P0,01), weight 5,3 kg (<P0,01), head circumference 39 cm (<P0,01) and she showed facial dysmorphisms (macroglossia, depressed nasal bridge, anteverted nares, hypertelorism, narrow palpebral fissures, low-set ears, short neck, low anterior hairline **(Figure 2)**), short limbs and excessive salivation. Medication included I-thyroxin, vigabatrin, melatonin, espusiman. She requires a platelet transfusion every 1-2 weeks. Additional investigations at 10 months of age yielded anemia (Hb 5,4 mmol/L; reference range 6,5-8,7 mmol/L), thrombocytopenia (29x10^9^/L; reference range 200-500x10^9^/L) and high alkaline phosphatase (443 U/L; reference range 124-341 U/L). White blood cell counts, TSH, cortisol, electrolytes, lipids, kidney and liver function tests were normal.

**Patient 5**

A female subject, 7 years old, born at 35 weeks of gestation as the first child of parents of German and Russian descent, family history was unsuspicious. The delivery was induced due to preeclampsia (art. hypertension, edema, proteinuria), birth weight was 1550 g (<P2,5), Apgar scores unknown. Feeding by nasogastric tube and care at the NICU was necessary up to 6 weeks. She came to medical attention with a delay of motor milestones and muscle hypotonia at age of 8 months. She was treated by physiotherapy and attained sitting at age 14 months, walking aided at 24 months and walking unaided at 30 months. Cognitive development was severely impaired and by the age of 30 months she showed no active or passive speech development. Standardized developmental testing was not possible. Treatment included ergotherapy, logopedic and pedagogical support and an individual case worker in the Kindergarden. At age of 5,7 years, she was tested (ET 6-6-R) in the age range of 24-30 months and showed a developmental score between the risk range and severe developmental deficits. Diagnostically, autism spectrum disorder was ruled out. Currently she is attending a school with the special educational focus ‘mental development’.

Brain MRI at age 2 years showed a persistent cavum septum pellucidum. No other medical problems like seizures, cardiac, endocrinological, urological or gastrointestinal disturbances were recognized.

Diagnosis of NANS-CDG was made at age 6 years based on genetic testing and revealed bi-allelic compound heterozygous variants of *NANS*, ManNAc levels were not measured. Radiographic images at age 7 years showed vertebral plates with sclerosis, lumbar lordosis and metaphyseal widening of the upper limbs with sclerosis and irregularity. Growth velocity decreased within the first years of life and short stature with short limbs and trunk were present; at follow-up at age 7 years her height was 105,6 cm (<P0,01), weight 17,2 kg (P0,5) and head circumference 50 cm (P10). Facial dysmorphisms included a narrow-accentuated forehead, eyelid axes discreetly directed outward downward, fine dense head hair, long eyelashes, narrow mouth, lower lip dimples and a slightly hypoplastic nose **(Figure 2)**. Due to strabismus and hyperopia, a spectacle fitting was necessary. No special medication was required, the girl was fed vegetarian diet. White and red blood cell counts, liver and kidney function, lactate, electrolytes, lipids, TSH and cortisol were normal.

**Patient 6**

Female subject from Iceland, aged 13 years, born at term after an uncomplicated pregnancy and delivery, birth weight was 3000 g (P8), Apgar scores unknown. **There is no family history or** consanguinity **of note**. Skeletal dysplasia (genu varum) was present at birth. Facial dysmorphisms included prominent forehead, flat nose, and epicanthus. Early developmental milestones were delayed: age of complete head control at 4 months, age of unaided sitting at 8 months, age of unaided walking at 20 months. Cognitive evaluation at age 6 years revealed severe mental retardation (IQ 20-40) and the absence of speech development. She was diagnosed with autism spectrum disorder. Hypotonia, mild ataxia, myopathy, strabismus and nystagmus were reported during childhood. Cardiac anomalies, hearing abnormalities and gastrointestinal pathology were absent. Radiographic examination of the skeleton showed vertebral plates with abnormal sclerosis, small iliac wings, small iliac heads and neck, metaphyseal widening of the upper and lower limbs with sclerosis and irregularity, dysplastic knee joints and fibular overgrowth **(Figure 4**)**.** Short limbs were present. CT scan of the brain at age 11 years showed no abnormalities. Brain MRI was not performed. She was growth restricted, with height of 115 cm (<P0,01) and weight 20 kg (<P0,3) at age 8,5 years, head circumference not measured at this age. She was not on any medication. Diagnostic testing was conducted at age 10 years based on whole genome sequencing (WGS). At this time, biochemical phenotype showed an elevated ManNAc excretion in urine of 46 µmol/mmol creatinine (reference value ‘not reported’). She is able to walk unaided and attends a special school. White and red blood cell counts, liver and kidney function were normal, as were electrolytes, lipids, TSH and cortisol.

**Patient 7**

A male subject, 16 years old, born at term as the second child of non-consanguineous parents of Dutch descent after an uneventful pregnancy, with a birth weight of 3620 g (P48), Apgar scores unremarkable. Family history of mother included hyperhomocysteinemia. Short-term post-partum respiratory support was required. He showed dysmorphic facial features: prominent forehead, sunken nasal bridge, prominent mouth, far outlined teeth, thickened teeth gums, gothic palate and big ears.

Motor development was unremarkable; sitting achieved at 8 months of age and unaided ambulation at 18 months of age. At age 2 years, he first came to medical attention with a mild delay in speech and language skills, emotional and communication skills, as well as fine motor skills. Auditory and ophthalmologic functions were normal.

NANS-CDG diagnosis was made at age 11 years via WES which revealed bi-allelic compound heterozygous variants of *NANS*. Metabolic testing showed an elevated ManAC excretion in urine of 45 µmol /mmol creatinine (reference value ‘not reported’) at 12 years of age.

Skeletal survey at age 11 years showed less calcification of the vertebral plates, small iliac wings, dysplastic acetabula, small femoral heads and neck and subtle metaphsyeal widening of the upper and lower limbs with with sclerosing. Short limbs were present. Orthopedic surgery of the femur and tibia was performed at age 12 years. His medical history included difficulties with swallowing, eczema and recurrent infections, however, no hospital admission was required. No cardiac anomalies or other gastrointestinal issues were noted. He was diagnosed with autism spectrum disorder at age 12 years; with an IQ 60-75 (verbal 73, performance 58). Contact (eye contact, social interactions) causes difficulties. Developmental testing at the biological age of 12 years revealed a cognitive developmental age of 8 years and a social-emotional age of 6 years according to ESSEON-R score.

Brain MRI at age 12 years showed a cavum septum pellucidum and vergae **(Figure 3)**. For behavioral issues and attention deficit issues he was prescribed methylphenidate (for 1 year); his sleep improved on melatonin. His puberty onset fell within normal range. He was able to speak sentences of a few words and to write some words at 13 years, but problems with behavioral and social interaction continued.

On exam at age 16 years he showed short stature with height 151,3 cm (P0,3), weight 47,3 kg (P5) and head circumference 55 cm (P80). He is able to cycle, swim and to dress himself; he is able to write but slowly and without fluency. Laboratory studies at last follow up (16 years of age) showed low levels of LDL (1,49 mmol/L; reference range 1,61-3,37 mmol/L) and triglycerides (0,55 mmol/L; reference range 0,8-2,0 mmol/L). Electrolytes, lactate, TSH, FSH, liver and kidney function were normal, as were white and red blood cell counts. At age 16 years, patient commenced experimental sialic acid therapy.

**Patient 8**

Patient 5, a 17-year old boy, was born at term as the second child to parents of Danish origin, after an unremarkable pregnancy; birth weight was 4560 g (P97), Apgar scores 4; 5; 6. Family history included no specific issues or consanguinity. At birth, hypotonia was noted and IV saline due to low blood pressure was required. Remarkable facial features included hypertelorism, wide nasal bridge, synophrys and irregular teeth. Developmental milestones were slightly delayed in the first years: unaided walking was achieved at 19 months, delay in fine motor skills was reported and vocal development started at 3 years of age. Intensive clinical examination is limited since symptoms were relatively mild. Neurological workup revealed hypotonia (neuroimaging was not performed). Gastrointestinal pathology was absent. No cardiac anomalies or short limbs were present. Hearing and visual evaluation were normal. Mild intellectual disability (IQ of 56 at age 6 years, test unspecified) and oral dyspraxia were observed. Onset of puberty was age adequate. Skeletal imaging was not performed. At 13,5 years, his height was 161 cm (P44), weight 70 kg (P95) (obese) and head circumference not measured. He was attending school for children with special needs and had good friends at school. At last follow-up, he was not on any medication. Laboratory studies were not performed. At age 15 years, WES revealed compound heterozygous *NANS* variants. Metabolic testing at 17 years revealed a slightly elevated ManNAc excretion in urine (10 µmol/mmol creatinine; reference value ‘not detected’).

**Patient 9**

A 28-year old female born at term as a child to non-consanguineous parents of Dutch origin, after an uncomplicated pregnancy; birth weight was 3920 g (P82), Apgar scores 10; 10. Family history included Noonan-syndrome (two cousins). At birth, some dysmorphisms at feet and hips, short limbs and neonatal jaundice (no phototherapy was required) were noted. Facial dysmorphisms included hypertelorism, prominent eyebrows, sunken nasal bridge with upturned nasal tip, prominent tent-shaped mouth with tongue protrusion, epicanthus, frontal bossing and mild macrocephaly.

Developmental milestones were delayed already in the first years: absence of head and trunk control (with a preference for right side) at 9 months, rolling from side to side at 2 years, generalized hypotonia and lack of movements at 3 years and no head control or walking at 5 years. An intensive period of clinical examination followed. Neuroimaging performed between 0 and 15 years showed ventriculomegaly (9 months), cerebral atrophy with atrophy of the caudate nucleus (3 years), hydrocephalus (15 years) and a cavum septum pellucidum (after reassessment of MRI) **(Figure 3)**. These findings became clinically manifest since she developed mild absence seizures (no medication needed) with an abnormal EEG at 15 years. She never developed language and social interactions were limited. Scoliosis, small femoral head and neck and small epiphyses were reported in the period between 5 and 7 years of age; scoliosis surgery was performed at the age of 10 years. She suffered from recurrent infections, severe abdominal distention (requiring laxative treatment and special diet), excessive mucus production and a bladder emptying disorder (requiring catheters). No cardiac anomalies were noted. Auditory and ophthalmologic functions are normal. Her puberty onset was not reported.

Diagnosis was made at age 25 years based on WES and revealed homozygous *NANS* variants. ManNAc excretion in urine was not determined, but ManNAc in plasma was abnormally increased, measured by NGMS. At 26 years, her height was 126 cm (<P0,01), weight 30 kg (<P0,01), while the head circumference was above the normal range (58 cm (>P95)). Currently, she is living in a nursing home due to profound intellectual disability, psychomotor developmental delay and wheelchair-dependency. At last clinical follow-up, she was not on any pharmacotherapy (the special diet eliminated the need for laxative treatment). Available laboratory studies at 2 years showed thrombocytopenia 50x10^9^/L (no reference value reported), recovered at age 28 years (154 x10^9^/L, reference 150-400x10^9^/L) and elevated lactate (4,2 mmol/L; reference 0,8-2,1 mmol/L). At 28 years iron deficiency anemia (due to special diet) (Hb 5,7 mmol/L, reference range 7,5-9,9 mmol/L) and a low LDL level (1,52mmol/L, reference range 1,76-4,09 mmol/L) were reported. At age 28 years, patient started with sialic acid experimental therapy. White cell counts, liver and kidney function, electrolytes, TSH and cortisol were normal.

**References**

1. van Karnebeek CD, Bonafé L, Wen XY, Tarailo-Graovac M, Balzano S, Royer-Bertrand B, et al. NANS-mediated synthesis of sialic acid is required for brain and skeletal development. Nat Genet. 2016;48(7):777-84.

.
